# Epigenetic regulation of white adipose tissue plasticity and energy metabolism by nucleosome binding HMGN proteins

**DOI:** 10.1038/s41467-022-34964-5

**Published:** 2022-11-26

**Authors:** Ravikanth Nanduri, Takashi Furusawa, Alexei Lobanov, Bing He, Carol Xie, Kimia Dadkhah, Michael C. Kelly, Oksana Gavrilova, Frank J. Gonzalez, Michael Bustin

**Affiliations:** 1 Protein Section, Laboratory of Metabolism, Center for Cancer Research, National Cancer Institute, National Institutes of Health, Bethesda, MD 20892 USA; 2 CCR Collaborative Bioinformatics Resource, Center for Cancer Research, National Cancer Institute, National Institutes of Health, Bethesda, MD USA; 3 Nucleic Acid Section, Laboratory of Metabolism, Center for Cancer Research, National Cancer Institute, National Institutes of Health, Bethesda, MD 20892 USA; 4 CCR Single Analysis Facility, Cancer Research Technology Program, Frederick National Laboratory for Cancer Research, Bethesda, MD 20892 USA; 5 Mouse Metabolism Core Laboratory, National Institute of Diabetes and Digestive and Kidney Diseases, National Institutes of Health, Bethesda, MD 20892 USA

**Keywords:** Epigenetics, Mechanisms of disease

## Abstract

White adipose tissue browning is a key metabolic process controlled by epigenetic factors that facilitate changes in gene expression leading to altered cell identity. We find that male mice lacking the nucleosome binding proteins HMGN1 and HMGN2 (DKO mice), show decreased body weight and inguinal WAT mass, but elevated food intake, WAT browning and energy expenditure. DKO white preadipocytes show reduced chromatin accessibility and lower FRA2 and JUN binding at *Pparγ* and *Pparα* promoters. White preadipocytes and mouse embryonic fibroblasts from DKO mice show enhanced rate of differentiation into brown-like adipocytes. Differentiating DKO adipocytes show reduced H3K27ac levels at white adipocyte-specific enhancers but elevated H3K27ac levels at brown adipocyte-specific enhancers, suggesting a faster rate of change in cell identity, from white to brown-like adipocytes. Thus, HMGN proteins function as epigenetic factors that stabilize white adipocyte cell identity, thereby modulating the rate of white adipose tissue browning and affecting energy metabolism in mice.

## Abstract (web summary)

White adipose tissue browning plays an important role in regulating whole-body energy homeostasis and metabolism. Here the authors show that the HMGN nucleosome binding proteins epigenetically promote white adipocyte differentiation and modulate the rate of white adipose tissue browning and energy metabolism in male mice.

## Introduction

Maintaining energy homeostasis, a balance between energy intake and energy expenditure is a fundamental requirement for survival and plays an important role in preventing metabolic disorders including obesity^1,2^. Adipose tissue facilitates energy homeostasis as it exists in two forms: white adipose tissues (WAT), enriched in cells that store energy as fat, and brown adipose tissue (BAT), enriched in adipocytes able to generate energy in the form of heat^2,3^. White adipocytes are plastic and under metabolic stress such as cold and fasting, they can convert into brown-like adipocytes a process known as “white adipocyte browning”^4,5^. At the molecular level, the browning of white adipocytes is facilitated by epigenetic factors such as DNA methylases, histone modifying enzymes, specific miRNAs, and transcription factors, which lead to changes in gene expression and altered cell identity^6,7^. Here we show that the chromatin-binding proteins high mobility group N (HMGN) modulate the rate of white adipocyte tissue browning.

HMGN is a family of ubiquitous chromatin-binding proteins that bind specifically to nucleosomes without any DNA sequence specificity. HMGNs are known to affect chromatin architecture, transcription factor binding, and gene expression in vertebrate cells^8–11^. Recent genome-wide and transdifferentiation studies revealed that HMGNs localize to cell-type-specific enhancer regions and stabilize cell identity^8^. Given the finding that HMGNs can function as epigenetic regulators that stabilize cell identity, we tested whether HMGNs affect white adipocyte browning and energy expenditure in mice.

In this work, we examined the role of HMGNs in white adipocyte browning by comparing wild-type (WT) mice and cells to genetically derived mice and cells lacking the two major members of the HMGN protein family, HMGN1 and HMGN2 (DKO mice). We find that DKO mice are smaller and show increased energy expenditure. Inguinal white adipose tissue (iWAT) from DKO mice has smaller adipocytes and characteristics suggesting increased adipocyte browning. Cultured white preadipocytes from DKO mice differentiate into brown-like adipocytes faster than white preadipocytes from WT mice, and DKO mouse embryonic fibroblasts (MEFs) transdifferentiate into brown-like adipocytes faster than WT MEFs. Transcription and epigenetic analyses of the differentiating cells link specific epigenetic changes, including altered epigenetic marks and chromatin binding of transcription factors, to accelerated white adipocyte browning in cells lacking HMGN1 and HMGN2 proteins. These findings identify HMGN proteins as epigenetic factors that affect energy metabolism by regulating the rate of iWAT browning.

## Results

### Enhanced white adipose tissue browning in HMGN DKO mice

**Fig. 1:**
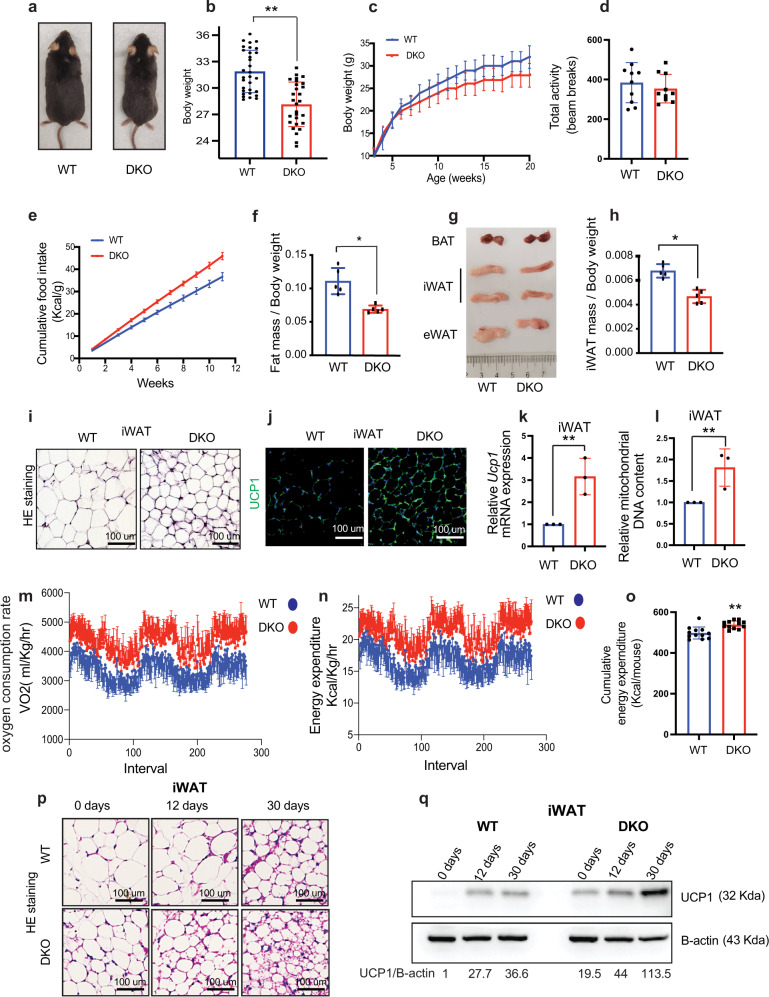
Enhanced white adipose tissue browning in HMGN DKO mice. **a** DKO mice are smaller than WT mice; shown are 25 weeks old WT and DKO mice. **b** Body weight of 25 weeks old WT and DKO mice (*n* = 28–31 for each group). **c** Body weight in growing WT and DKO mice (*n* = 12 for each group). **d** Total activity of WT and DKO mice, measured as beam breaks (*n* = 12 for each group). **e** Cumulative food intake of WT and DKO mice (*n* = 12 for each group). **f** Fat mass of WT and DKO mice. Tissue mass is normalized to body weight (*n* = 5 for each group. Data are mean ± SD; *p = 0.0135). **g** Gross appearance of adipose tissues from WT and DKO mice. **h** iWAT weight of WT and DKO mice. Tissue mass normalized to body weight (*n* = 5 for each group. Data are mean ± SD; *p = 0.005). **i** Histology of WT and DKO iWAT. **j** UCP1 immunostaining of WT and DKO iWAT. **k** Quantitative RT-PCR analysis of *Ucp1* expression in iWAT from WT and DKO mice (*n* = 3 for each group). **l** Quantitative RT-PCR analysis of mitochondrial DNA content in WT and DKO iWAT (*n* = 3 for each group). **m** Elevated oxygen consumption rate in DKO mice measured by indirect calorimetry. **n** Elevated energy expenditure in DKO mice measured by indirect calorimetry. **o** Elevated cumulative energy expenditure in DKO mice measured by energy balance (*n* = 12 for each group). **p** Histology of iWAT from intermittent fasting WT and DKO mice. **q** Western blot showing UCP1 expression in iWAT of intermitted fasting WT and DKO mice. UCP1/B-actin values are shown below each column. Data are the mean ± SD. A two-tailed *t*-test was used in (**b**, **f**, **h**, **k**, **l**, **o**); *p < 0.05, and **p < 0.005 are considered significant over control. For **i**, **j**, **p**, and **q** data are representative of two independent experiments with similar results. All measurements were done with age-matched, WT and DKO male mice.

DKO mice fed a standard chow diet showed decreased body weight throughout development, and adult DKO mice exhibited a 10% decrease in body weight compared to age-matched, control WT mice (Fig. 1a–c). The decrease in body weight was not linked to increased total physical activity or decreased food intake since measurements in activity chambers did not show a difference between WT and DKO mice in total activity (Fig. 1d), and DKO mice showed increased food intake (Figs. 1e, S1a and Supplementary Data 1). Magnetic resonance imaging (MRI) analyses revealed that the decrease in body weight was associated with a significant reduction in the total fat mass of the DKO mice (Fig. 1f), but the total lean mass was not altered significantly (Fig. S1b). DKO mice had significantly reduced amount of inguinal WAT (iWAT) (Fig. 1g, h) but both their epididymal WAT (eWAT) and brown adipose tissue (BAT) were not significantly different from that of WT mice (Figs. 1g, S1c, d). Hematoxylin and eosin (H&E) staining revealed that iWAT adipocytes from DKO mice are markedly smaller than WT adipocytes (Figs. 1i, S1e). In addition, both immunofluorescence and quantitative RT-PCR indicate higher levels of mitochondrial uncoupling protein 1 (UCP1) expression (Fig.1j, k), a significant upregulation of transcripts encoding additional thermogenic markers (Fig. S1f), and a 2-fold increase in mitochondrial DNA in DKO iWAT (Fig. 1l). The eWAT of WT and DKO mice showed equal levels of *Ucp1* RNA (Fig. S1g). We also observed that the expression of *Hmgn1* and *Hmgn2* mRNAs was considerably higher in iWAT than in eWAT, (Fig. S1h). An indirect calorimetry experiment revealed increased oxygen consumption (Fig. 1m) and increased energy expenditure (Fig. 1n) in DKO mice. Cumulative energy expenditure measurements by energy balance techniques also indicated increased energy expenditure in DKO mice (Figs. 1o, S1i, and Supplementary Data 1b). Taken together, these results suggest elevated WAT browning and energy expenditure in DKO mice.

Intermittent fasting promotes iWAT browning in mice^12^ leading to increased energy expenditure in part due to higher levels of mitochondria and UCP1 protein^13^. Intermittent fasting of WT and DKO mice fed a standard chow diet every other day, induced the browning of WAT in DKO mice to a significantly higher level than in WT mice, as seen by beige adipocytes with multilocular morphology in H&E staining (Figs. 1p, S1j) and elevated UCP1expression seen by western blot analyses (Fig. 1q), and by immunostaining (Fig. S1k). BAT did not show differences between WT and DKO mice (Fig. S2) suggesting that the increased energy expenditure in DKO mice is mainly due to enhanced WAT browning.

In summary, mice lacking HMGN1 and HMGN2 show reduced body weight and fat mass but increased food intake, elevated energy expenditure, and enhanced rate of iWAT browning. These results raise the possibility that HMGN proteins affect white adipocyte browning and energy expenditure in mice.

### DKO mice fed high-fat diet (HFD) show enhanced white adipose tissue browning

**Fig. 2:**
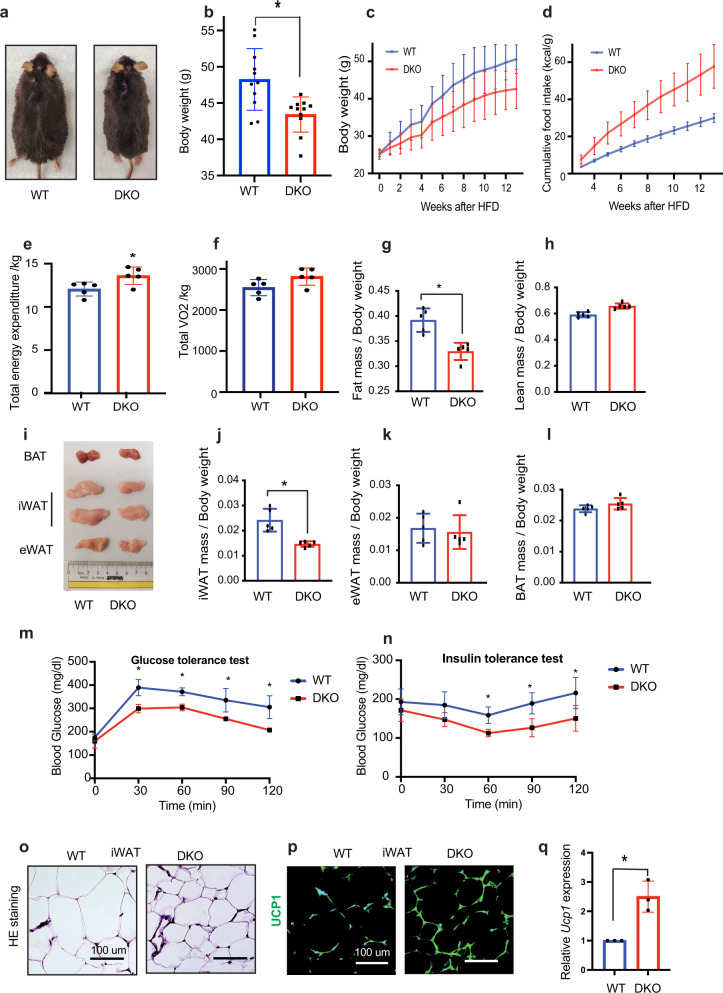
Enhanced white adipose tissue browning in HFD-fed DKO mice. **a** Gross appearance of representative WT and DKO mice fed with HFD for 16 weeks. **b** Body weight of WT and DKO mice fed HFD for 16 weeks. **c** Body weight of WT and DKO mice during HFD feeding for 12 weeks (*n* = 12 for each group). **d** Cumulative food intake of WT and DKO mice fed with HFD for 12 weeks (*n* = 12 for each group). **e** Elevated total energy expenditure in DKO mice during HFD feeding (*n* = 5 for each group). **f** Total VO2 in WT and DKO mice during HFD feeding (*n* = 5 for each group). **g** Fat mass of WT and DKO mice. Tissue mass normalized to body weight (*n* = 5 for each group). **h** Lean mass of WT and DKO mice. Tissue mass normalized to body weight (*n* = 5 for each group). **i** Gross appearance of adipose tissues from WT and DKO mice. **j** iWAT weight of WT and DKO mice (*n* = 5 for each group). **k** eWAT weight of WT and DKO mice fed with HFD for 16 weeks (*n* = 5 for each group). **l** BAT weight of WT and DKO mice fed with HFD for 16 weeks (*n* = 5 for each group). **m** Glucose tolerance test performed on WT and DKO mice fed with HFD for 16 weeks (*n* = 5 for each group). **n** Insulin tolerance test performed on WT and DKO mice (*n* = 5 for each group). **o** Histology of iWAT from WT and DKO mice. **p** UCP1 immunostaining of iWAT from WT and DKO mice. **q** Quantitative RT-PCR analysis of *Ucp1* expression in iWAT. All data from male WT and DKO mice fed HFD for 16 weeks. For **o** and **p**, data are representative of two independent experiments with similar results. Data are the mean ± SD. A two-tailed *t*-test was used in (**b**, **e**, **g**, **j**, **m**, **n**, and **q**); *p < 0.05.

Compared to HFD-fed WT mice, DKO mice fed HFD for 16 weeks, gained considerably less weight and appeared smaller (Fig. 2a–c), and showed elevated food intake (Fig. 2d), and energy expenditure (Fig. 2e), but VO2 (Fig. 2f) was not significantly altered. HFD-fed DKO mice had lower fat mass, but not lean mass, as determined by MRI analyses (Fig. 2g, h). Likewise, these mice show reduced iWAT size and weight (Fig. 2i, j), but no change in eWAT or BAT (Fig. 2i, k, l). In addition, both glucose and insulin tolerance tests showed improved glucose metabolism in HFD-fed DKO compared to control, WT mice (Fig. 2m, n). H&E staining of the iWAT from HFD-fed DKO mice reveals smaller adipocytes (Fig. 2o) and both immunostaining and transcription analyses of these mice show increased UCP1 expression (Fig. 2p, q). Thus, loss of HMGN enhanced the rate of iWAT browning in mice fed either standard chow or HFD.

### Loss of HMGN enhances the rate of white preadipocyte browning

**Fig. 3:**
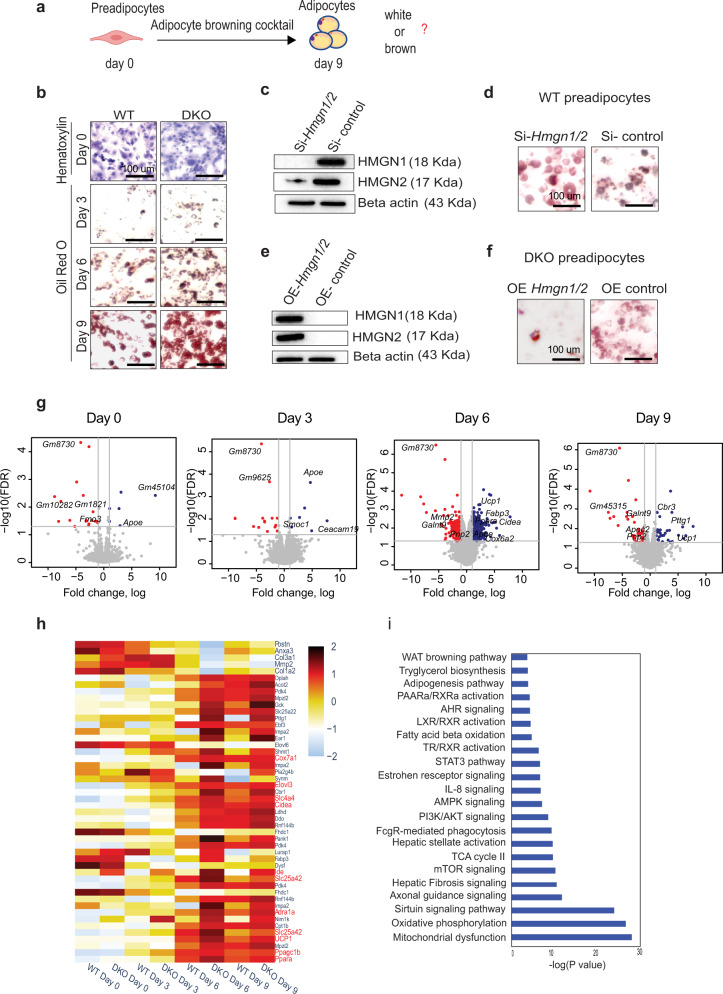
Enhanced browning efficiency in DKO white preadipocytes. **a** Model of In vitro differentiation of white preadipocytes. Created with BioRender.Com. **b** Oil Red O staining of WT and DKO adipocytes differentiated from white preadipocytes. 0 day were stained with hematoxylin; 3rd, 6th, and 9th day were stained with Oil Red O. **c** Western blot analysis of HMGN1 and HMGN2 expression in WT preadipocytes transfected with *Hmgn1* and *Hmgn2* siRNA or control, scrambled siRNA. **d** Oil Red O staining of in vitro differentiated WT adipocytes transfected with *Hmgn1* and *Hmgn2* siRNA or control, scrambled siRNA. **e** Western blot analysis of HMGN1 and HMGN2 expression in DKO preadipocytes transfected with a plasmid expressing either *Hmgn1* and *Hmgn2* or scrambled control. **f** Oil Red O staining of in vitro differentiated DKO adipocytes transfected with *Hmgn1* and *Hmgn2* expression plasmids or control plasmids. **g** Volcano plots showing differential gene expression (fold change ≥ .5; FDR < 0.05) between WT and DKO adipocytes differentiated from preadipocytes. A list of all the differentially expressed genes is shown in supplementary dataset 2. **h** Heatmap showing enhanced upregulation of genes associated with white adipocyte browning during DKO preadipocyte differentiation, compared to WT. **i** IPA analysis of preferentially regulated pathways in DKO adipocytes during the 6th day of adipocyte differentiation. Data are from three biological replicates.

To verify that loss of HMGNs is sufficient to enhance the rate of adipocyte browning, we isolated iWAT preadipocytes from WT and DKO mice and induced differentiation using an adipogenic cocktail known to facilitate differentiation into brown-like adipocytes (Fig. 3a)^14^. Oil-Red O staining and qRT-PCR analysis of the in vitro differentiated adipocytes revealed that DKO preadipocytes differentiate into mature beige adipocytes faster than WT preadipocytes (Figs. 3b and S3a). Quantitative analyses by ImageJ software indicate 2-fold higher levels of lipid-containing cells in the differentiating adipocytes derived from DKO mice (Fig. S3b). These systemic effects are indeed due to loss of HMGNs since si-RNA mediated downregulation of HMGN in wild-type preadipocytes (Fig. 3c) enhanced adipogenesis (Figs. 3d, S3c) while the rescue of HMGN expression in DKO preadipocyte by vectors expressing HMGN1 and HMGN2 (Fig. 3e) inhibited adipogenesis (Figs. 3f, S3d). RNA-seq analyses during in vitro adipocyte differentiation showed differences in gene expression between WT and DKO cells (Fig. 3g and Supplementary Data 2). Throughout differentiation the levels of HMGNs varied (Fig. S3f) and genes known to be expressed in brown adipocytes such as *Ucp1*, *Ppargc1a*, *Cidea, Elovl3*, *Cox7a1*, and *Ear2* were upregulated in DKO cells, as compared to WT cells (Figs. 3h and S3e), while non-adipogenic genes such as *Col1a2*, *Col3a1*, *mmp2*, *mmp3*, *Anxa3* were reduced in DKO adipocytes (Fig. 3h). Ingenuity Pathway Analysis (IPA) of the RNA-seq data at day 6 of differentiation revealed that pathways known to affect WAT browning, fatty acid β-oxidation, TCA cycle, and oxidative phosphorylation were among the most upregulated in DKO adipocytes (Fig. 3i). In summary, white preadipocytes lacking HMGNs differentiated into brown-like adipocytes more efficiently than WT white preadipocytes. Furthermore, DKO preadipocytes also showed enhanced efficiency in differentiation into mature white adipocytes when differentiation was induced with a general adipogenic cocktail (Fig. S3g).

### Single-cell analysis of white preadipocytes differentiation into brown-like adipocytes

**Fig. 4:**
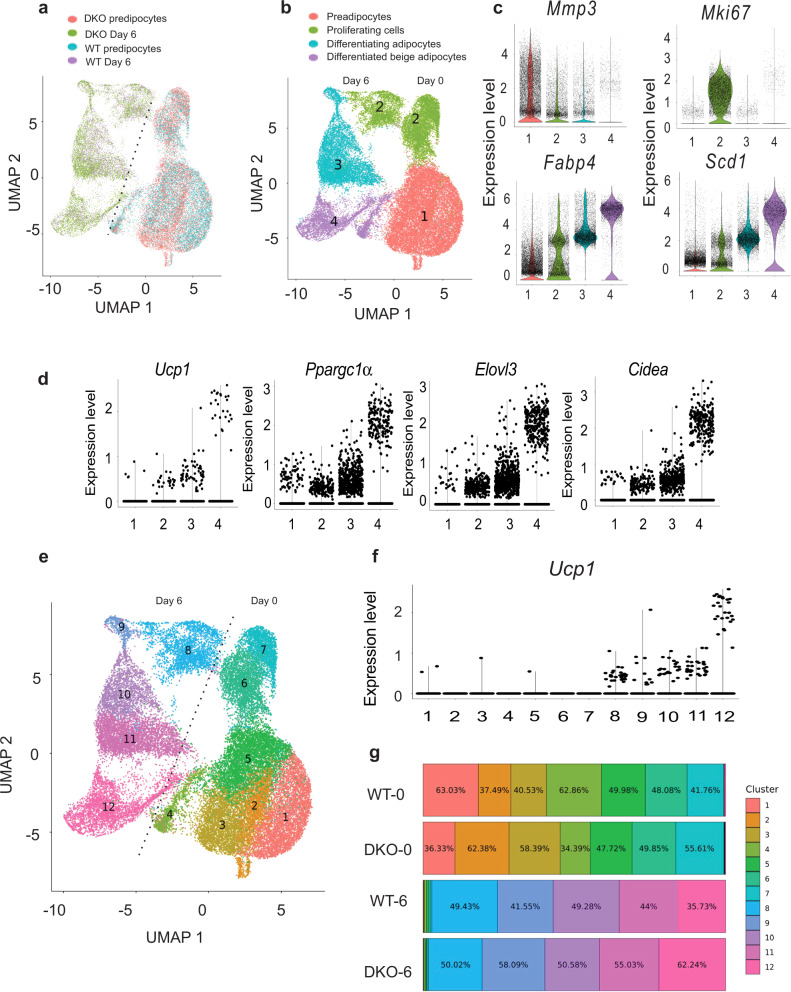
Single-cell RNA seq analysis during white preadipocyte browning. **a** UMAP plot identifying the population of WT or DKO preadipocytes in the separated cell clusters at days 0 and 6 of induced browning. Duplicate samples of white preadipocytes derived from either WT or DKO mice were induced to differentiate. Samples were collected at day 0 and day 6 and analyzed by scRNA seq. Shown is an aggregate plot of all samples. **b** UMAP plots showing various annotated clusters of aggregate scRNAseq analysis at days 0 and 6 during the browning of white preadipocytes. **c** Expression level of genes in various cell clusters shown in (**b**). Numbers on the *x*-axis correspond to cell clusters shown in panel (**b**). **d** Expression level of brown adipocyte-specific genes in various cell clusters shown in (**b**). **e** High-resolution UMAP plot of WT and DKO white preadipocytes at day 0 and day 6 of induced browning. **f** Expression level of *Ucp1* in different clusters identified in panel (**e**). **g** Percentage of cell fractions of each cell cluster shown in panel **e**, in WT and DKO cells, at day 0 and day 6 of induced browning.

To further verify that loss of HMGN enhances the rate of white adipocyte browning and explore the possibility that DKO white preadipocytes contain unique populations that facilitate browning, we performed single-cell RNA-seq (scRNA) on WT and DKO white preadipocytes at day 0 and day 6 of induced browning. Aggregate analyses of the differentiating WT and DKO iWAT preadipocytes show a clear separation between preadipocytes clusters at day 0 and clusters of differentiating adipocytes at day 6 (Fig. 4a). WT and DKO preadipocytes are intermingled and evenly distributed over the two preadipocyte clusters, while the cell clusters associated with differentiated adipocytes are enriched in DKO cells (note green color in Fig. 4a). We performed graph-based clustering of cells (Figs. 4b and S4d) and based on the cluster-enriched expression of genes we annotated cluster 1 as preadipocytes (*Mmp3, Cd142*, and *Itgb1*) (Figs. 4c, S4a)^15–18^, cluster 2 as proliferating cells (*Mki67*), while cluster 3 as differentiating adipocytes (*Col5a3, Serpina3n*) (Fig. S4b)^19^ and cells in cluster 4 as differentiated beige adipocytes (*Ucp1*, *Ppargc1a, Elovl3, Cidea*) (Figs. 4d and S4c). We also observed the statistical significance of thermogenic genes among different clusters between the two genotypes investigated (Supplementary Data 3). Higher-resolution clustering resulted in 12 clusters, with cluster 12 containing cells with the highest expression of mature brown adipocyte marker *Ucp1* (Fig. 4e, f). Consistent with the loss of HMGN increases the rate of adipocyte browning, clusters 9,11, and 12 showed increased presence of DKO cells (Figs. 4g and S4e); cluster 12 contained a higher proportion of DKO cells (60%) compared to day 6 WT adipocytes (40%) (Fig. S4e). The major genes in 12 clusters are shown as a heatmap in Fig. S5. Trajectory analysis was performed with cells showing more mature brown adipocyte phenotype markers such as *Ppargc1a*, *Cidea*, and *Elovl3* later in pseudo time ordering (Fig. S4f, g). Cells from day 6 DKO samples contributed to a higher percentage of the cells in the most mature brown-like adipocyte cell state (cell state 6) in the trajectory analysis. In the preadipocyte clusters (Fig. 4g), we note clusters enriched in DKO cells (clusters 2,3) and clusters enriched in WT cells (clusters 1,4) which is consistent with previous studies showing that loss of HMGNs alters the cellular transcription profile^20,21^. Likely, the HMGN-dependent alterations in the transcription profiles of white preadipocytes lead to enhance the rate of DKO adipocyte browning.

In summary, both mRNA and scRNA analyses indicate that loss of HMGNs enhanced the rate of white preadipocyte browning.

### HMGN proteins affect chromatin accessibility and transcription factor occupancy in white preadipocytes

**Fig. 5:**
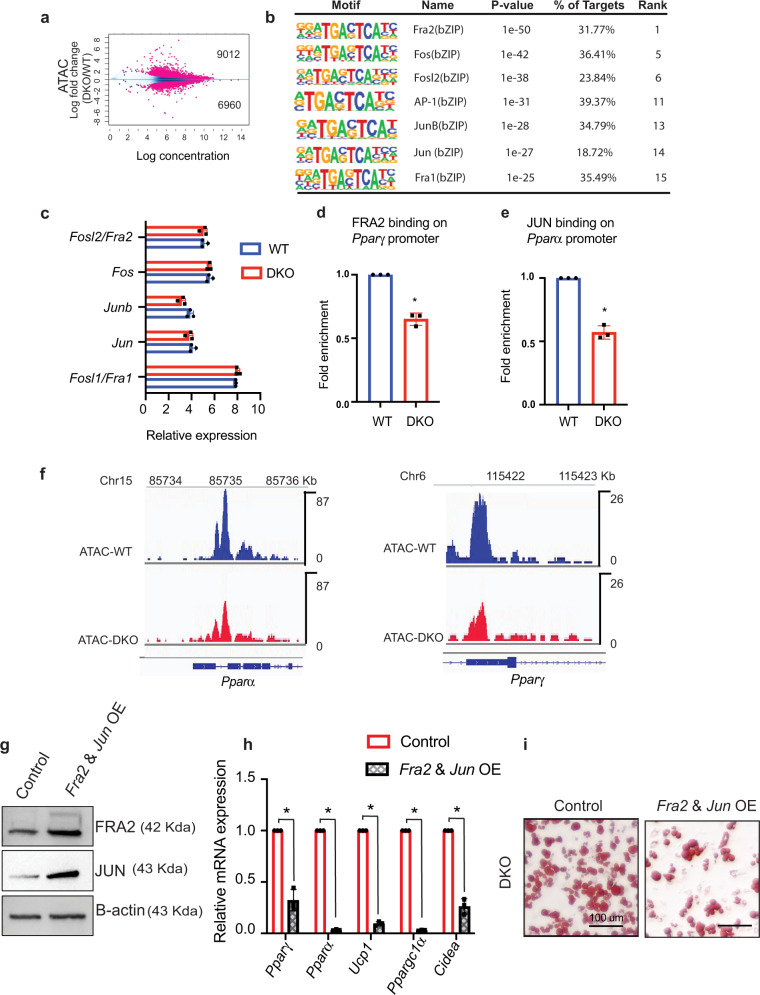
HMGN affects chromatin accessibility and transcription factor chromatin binding in adipocytes. **a** MA plot showing differences in ATAC peaks between WT and DKO preadipocytes. A list of all the significantly altered ATAC regions is shown in Supplementary Data 4. **b** Loss of ATAC sensitivity at AP-1 transcription factor binding motifs. **c** Similar levels of AP-1 transcription factor transcripts in WT and DKO preadipocytes. Data are from three biological replicates. **d** ChIP quantitative RT-PCR analysis showing decreased binding of FRA2 to *Pparγ* promoter in DKO preadipocytes. Data are the mean ± SD; *n* = 3 for each group. *p = 0.0064. **e** ChIP quantitative RT-PCR analysis showing decreased binding of JUN binding at *Pparα* promoter in DKO preadipocytes. Data are the mean ± SD; *n* = 3 for each group. *p = 0.0049. Data are from three biological replicates. **f** IGV plots showing decreased ATAC sensitivity at *Ppar* genes of WT and DKO preadipocytes. **g** Western blot showing elevated levels of FRA2 and JUN protein levels in DKO cells transfected with Fra2 and Jun mammalian expression vectors. **h** Quantitative RT-PCR analysis of *Pparγ* and *Pparα* and brown adipocyte-specific genes *Ucp1*, *Ppargc1a*, and *Cidea* in vitro differentiated DKO adipocytes transfected with either control or with Fra2 and Jun mammalian expression vectors. **i** Upregulation of FRA2 and JUN in adipocytes inhibits browning. Shown are Oil Red-O staining of day 9 in vitro differentiated DKO adipocytes transfected with either control or with Fra2 and Jun mammalian expression vectors. Note decreased Oil Red-O staining in the cells overexpressing FRA2 and JUN. Data are the mean ± SD; *n* = 3 for each group. A two-tailed *t*-test was used in (**d**, **e**, and **h**); *p < 0.05.

Changes in gene expression often involve changes in chromatin organization and chromatin accessibility^22^. We, therefore, performed a genome-wide assay for transposase-accessible chromatin sequencing (ATAC-seq)^23^ in white preadipocytes isolated from WT and DKO mice. MA plot shows numerous statistically significant changes in chromatin accessibility between WT and DKO (Fig. 5a and Supplementary Data 4) raising the possibility that in DKO preadipocytes, loss of HMGN altered chromatin accessibility and the binding of regulatory factors to chromatin^10,11^. A query for transcription factor binding motifs at sites that showed reduced ATAC sensitivity in DKO preadipocytes revealed that 7 out of the top 15 motifs serve as binding sites for the AP1 group of transcription factors such as FRA2, FOS, JUN, JUNB, and FRA1 (Fig. 5b, Supplementary Data 5). Transcription analysis revealed equal expression levels of all these regulatory factors in WT and DKO cells (Fig. 5c), raising the possibility that HMGNs affect the ATAC sensitivity of these sites by affecting the binding of these regulatory factors to chromatin.

We focused on FRA2 and JUN since they are known to inhibit white adipocyte browning by suppressing the expression of *Pparγ* and *Pparα*; two factors known to promote adipocyte browning^24–26^. Chromatin immunoprecipitation analysis revealed decreased binding of FRA2 and JUN at *Pparγ* and *Pparα* promoter regions, respectively (Fig. 5d, e) and ATAC analysis showed decreased chromatin accessibility of *Pparγ* and *Pparα* genes in DKO cells (Fig. 5f). Consistent with previous findings that FRA2 and JUN are negative regulators of *Pparγ* and *Pparα* gene expression^24,25^, the decreased binding of Fra2 and Jun to *Pparγ* and *Pparα* promoters in DKO preadipocytes resulted in elevated expression of *Pparγ* and *Pparα* during white adipocyte browning as seen by RNA seq (Fig. S6a) and qRT-PCR (Fig. S6b).

To further verify that the enhanced adipocyte differentiation and browning seen in DKO adipocytes is linked to FRA2 and JUN, we transfected DKO preadipocytes with vectors expressing FRA2 and JUN or with control vectors (Fig. 5g). We find that upregulation of FRA2 and JUN lead to decreased expression of PPAR*γ* and PPAR*α*, downregulation of thermogenic genes such as *Ucp1*, *Ppargc1a* and *Cidea* (Fig. 5h) and decreased oil red staining (Fig. 5i). These findings agree with our IPA analysis of RNA seq data (Fig. 3i) which also indicated upregulation of PPARα activation and WAT browning pathways in DKO cells. Given the previous findings that elevated expression of *Pparγ* and *Pparα* and their target genes, such as *Pgc1a* and *Ucp1*, facilitate white adipocyte browning^26–28^, these results indicate that HMGN proteins modulate the rate of adipocyte browning by epigenetically regulating the expression of *Pparγ* and *Pparα*. Taken together, the results provide further support for the notion that HMGN-mediated reduction of FRA2 and JUN chromatin binding leads to elevated PPAR*γ* and PPAR*α* expression, upregulated thermogenic gene expression, and enhanced adipocyte browning.

### HMGN proteins modulate epigenetic landscape during white adipocyte browning

**Fig. 6:**
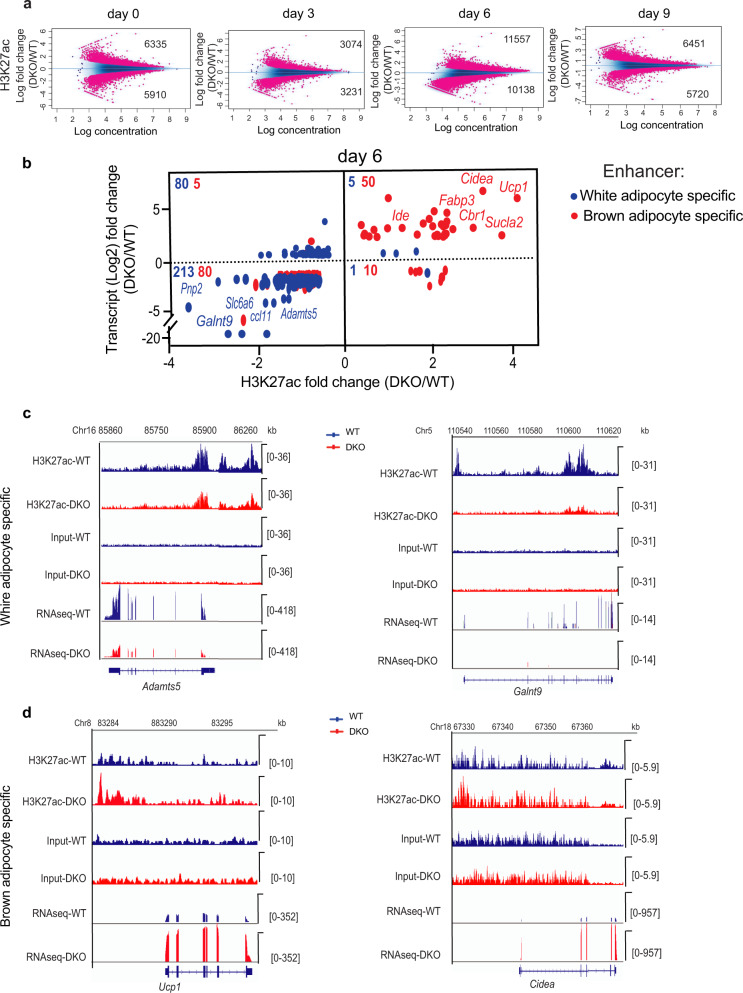
HMGNs affect H3K27ac levels and adipocyte type-specific gene expression during the browning of white preadipocytes. **a** MA plot showing differences in H3K27 acetylation between WT and DKO preadipocytes induced browning at 0-day, 3rd day, 6th day, and 9th day. A list of all the significantly altered H3K27ac regions is shown in supplementary data 6. Data are from three biological replicates. **b** Correlation plot of H3K27ac with transcript levels at white and brown specific enhancers. The red and blue numbers in the upper left of each quadrant indicate the number of white and brown adipocyte-specific genes. **c**, **d** IGV screenshots showing H3K27ac and transcript levels at white (**c**) and brown-specific enhancers (**d**) at day 6 of preadipocyte browning.

Transcriptional changes during white adipocyte browning are associated with changes in the epigenetic landscape, especially histone acetylation of H3K27 at cell-type-specific enhancers^29–31^. Given that the HMGN proteins colocalize with H3K27ac and modulate the levels of H3K27ac at cell-type-specific enhancers and stabilize cell identity^8,32,33^, we tested whether HMGNs alter H3K27ac levels during white preadipocyte differentiation into brown-like adipocytes. ChIP analyses of cultured white preadipocytes showed numerous differences in acetylation levels between WT and DKO preadipocytes at day 0 and 6 of preadipocyte differentiation (Fig. S7a) and MA plots showed numerous statistically significant global changes in H3K27ac levels between WT and DKO during the entire course of differentiation (Fig. 6a and Supplementary Data 6).

We then focused on known white and brown adipocyte enhancers and their associated genes^34^ and examined their H3K27ac and transcription levels during the induced browning of white preadipocytes from WT and DKO mice. We found that 213 white, but only 80 brown adipocyte-specific genes were preferentially downregulated and also showed decreased H3K27ac levels in DKO cells (Fig. 6b). Conversely, 50 brown adipocyte-specific enhancers but only 5 white-specific enhancers showed preferential upregulation of both transcript and H3K27ac levels in DKO cells (Fig. 6b). Among the genes that were most downregulated and showed the highest loss of H3K27ac in DKO cells were white adipocyte-specific genes such as *Adamts5 Galnt9, Slc6a6*, and *Pnp2* (Fig. 6b, c) and among the most upregulated genes were brown-specific genes such as *Ucp1*, *Cidea*, and *Ide* (Fig. 6b, d). Consistent with the increased expression observed, *Pparα* promoter possessed elevated H3K27 acetylation in Day 6 DKO cells, while *Pparγ* promoter has equal levels of acetylation in both the genotypes studied (Fig. S7b). The list of transcripts and their associated white and brown specific enhancers that are significantly altered for all the time points analyzed are shown in Supplementary Data 7.

In summary, the data revealed a genotype-specific correlation between gene expression and H3K27ac levels during adipocyte browning. DKO cells show elevated H3K27ac levels and gene expression of brown adipocyte-specific genes but decreased H3K27ac and transcription levels of white adipocyte genes. The changes in H3K27ac levels, together with the changes in gene expression suggest that loss of HMGN accelerated the loss of white adipocyte cell identity during browning.

### Loss of HMGN enhances the differentiation rate of mouse embryonic fibroblasts (MEF) into brown-like adipocytes

As an additional test that HMGN proteins can modulate adipocyte browning, we differentiated mouse embryonic fibroblasts (MEFs) from WT and DKO mice into adipocytes using a cocktail known to facilitate differentiation into brown-like adipocytes^14,35,36^ (Fig. S8a). Oil Red O staining of the differentiating cells indicates that DKO MEFs differentiated into adipocytes faster than WT MEFs (Fig. S8b, c). Similar to the preadipocyte differentiation experiments, we confirmed that the effects are indeed due to loss of HMGNs, since si-RNA mediated downregulation of HMGN in WT MEFs (Fig. S8d) enhanced adipogenesis (Fig. S8e, h) while the rescue of HMGN expression in DKO MEFs with vectors expressing HMGN1 and HMGN2 (Fig. S8f) inhibited adipogenesis (Fig. S8g, i). RNA-seq analyses of the MEFs collected at several time points during differentiation showed differences in gene expression between WT and DKO cells (Fig. S8j, k and Supplementary Data 8). Genes such as *Pparγ*, *Pparα*, and *Cidea* and brown adipocyte-specific genes (*Ucp1*, *Ppargc1b*, *Elovl3, Adrb3*) were upregulated in DKO cells compared to WT cells, confirming that DKO MEF cells differentiated into brown-like adipocytes faster than WT MEFs. IPA analysis of the genes differentially expressed between WT and DKO adipocytes 12 days after onset of differentiation revealed that signaling pathways related to adipogenesis, such as WAT browning, adipogenesis, PPARα activation, oxidative phosphorylation, and mitochondrial dysfunction pathways were elevated in DKO adipocytes (Fig. S8l). The enhanced differentiation of DKO MEFs into brown-like adipocytes supports the role of HMGN proteins in modulating the rate of white adipocyte browning.

## Discussion

The plasticity of adipose tissue is known to play a role in maintaining energy homeostasis and affect metabolic processes. Thus, white adipose tissue browning facilitates energy expenditure, raising the possibility that it may reduce obesity and improve metabolic health^37,38^. However, excessive WAT browning is also associated with adverse outcomes such as cachexia and atherosclerosis^39^. Understanding the mechanisms that affect the plasticity of adipocyte cell lineage identity may contribute to elucidating the molecular mechanism involved in regulating metabolic processes that facilitate the proper maintenance of energy homeostasis.

Changes in adipose tissue phenotypes, including iWAT browning, are driven by factors that facilitate changes in gene expression and lead to an altered cell identity^6,40,41^. It is well documented that the maintenance of cell identity is critically dependent on the dynamic nature of the epigenetic landscape encoded in chromatin^42,43^. The chromatin epigenetic landscape needs to be sufficiently stable to prevent deleterious changes in gene expression yet sufficiently plastic to allow a proper response to the metabolic events that lead to physiological advantageous changes in cell identity. The chromatin landscape is established and maintained by nuclear components known to regulate chromatin dynamics and gene expressions such as factors that affect the level of histone or DNA modifications, nucleosome organization, chromatin topology, and compaction. Here we showed that the nucleosome-binding proteins HMGN1 and HMGN2, which are known to modulate chromatin compaction^44^ and gene expression^45^ affect energy expenditure and WAT browning in mice and in cultured cells.

Several experimental observations support the link between HMGN proteins and WAT browning. First, experiments with mice fed either chow or a high-fat diet reveal that the DKO mice show reduced body weight and increased iWAT browning, and chow-fed DKO mice showed increased energy expenditure. Second, in vitro differentiation of DKO white preadipocytes shows an enhanced rate of browning and an elevated rate of brown adipocyte-specific gene expression. Our in vitro experiments with siRNA-mediated knockdown of *Hmgn* in WT cells and overexpression of *Hmgn* in DKO cells strongly supported the in vivo effects seen by the loss of HMGN proteins. Third, H3K27ac ChIP seq and RNA-seq correlation analysis reveal elevated H3K27ac levels at brown adipose-specific enhancers but decreased H3K27ac levels at white adipose-specific enhancers in DKO cells. Fourth, the differentiation of WT and DKO MEFs into adipocyte-like cells shows increased browning and increased brown adipose-specific gene expression in DKO MEFs. Although all experiments were done with male mice it is likely that similar mechanisms are operative in female mice.

**Fig. 7:**
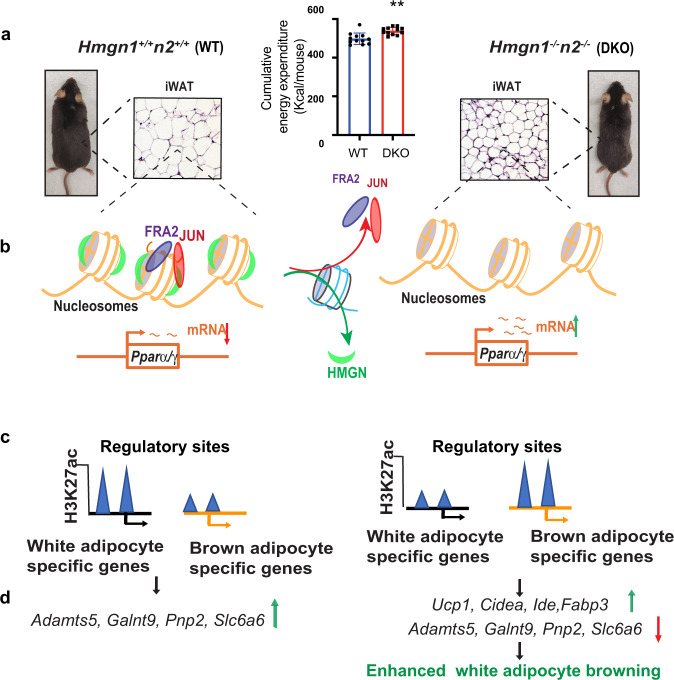
Model summarizing the effect of HMGNs on white adipocyte browning. **a** Mouse phenotype DKO mice are smaller, show decreased iWAT cell size, and elevated energy expenditure. Data are the mean ± SD; *n* = 3 for each group. *p < 0.05. **b** Altered binding of transcription regulators. Loss of HMGN decreases chromatin accessibility and the binding of FRA2 and JUN at *Ppar*a/g promoters and enhances *Ppar*a/g transcription. **c** Altered epigenetic landscape. DKO mice show decreased H3K27ac levels at enhancers of white adipocyte-specific genes but increased H3K27ac levels at enhancers of brown adipocyte-specific genes. **d** Altered transcription. Loss of HMGNs leads to changes in gene expression and to an enhanced rate of white adipocyte browning.

HMGNs reduce chromatin compaction^44^ and bind dynamically to nucleosomes without DNA sequence specificity; however, they preferentially localize to enhancers and promoters, chromatin regulatory regions that are marked by high levels of H3K27ac. HMGNs modulate chromatin dynamics during cellular differentiation^9,46^ most likely because they affect the binding of regulatory factors to chromatin^10^, and regulate cell-type-specific gene expression^11,45^. We now find that in white preadipocytes, loss of HMGNs reduces the chromatin accessibility and chromatin binding of the known negative regulators FRA2 and JUN to the promoter regions of *Pparγ* and *Pparα*, two master transcription factors known to enhance white fat browning^27,28^. In agreement, loss of HMGNs enhances *Pparγ* and *Pparα* expression during white preadipocyte browning, elevates brown adipocyte gene expression, and enhances the rate of white preadipocyte browning. A model summarizing the postulated mechanism whereby HMGNs affect the rate of white adipose tissue browning and energy metabolism in mice is shown in Fig. 7.

While other transcription factors might also contribute to increased browning in DKO white preadipocytes, we postulate that decreased binding of the two negative regulators FRA2 and JUN to *Pparγ* and *Pparα* gene promoters in DKO white preadipocytes leads to increased expression of *Pparγ* and *Pparα* and to enhanced rate of browning in DKO cells. Thus, HMGNs epigenetically stabilize white adipocyte cell identity and regulate the browning of white preadipocytes by modulating transcription factor binding and H3K27ac levels at adipocyte-specific enhancers. Our findings highlight an additional mechanism that affects white adipocyte browning and regulates energy metabolism in mice. Given the ubiquitous presence of HMGNs in all vertebrate cells it is likely that similar mechanisms are operative in other vertebrates.

## Methods

### Mice and ethics statement

*Hmgn1*^*−/−*^ and *Hmgn2*^*−/−*^ mice (DKO mice) were generated in our laboratory as described^47^. The WT and DKO mice and cells derived from these mice have been previously extensively characterized^8,10,47^. DNA sequence analyses show the absence of the genomic sequences deleted, RNA sequence analyses show the absence of transcripts of the deleted exons, and western analyses show that DKO cells do not express HMGN1 and HMGN2 proteins. All animal experiments complied with the protocols for animal use, treatment, and euthanasia approved by the National Cancer Institute Institutional Animal Care and Use Committees (animal protocols: LMC-030 and LM-096). *Hmgn1*^*−/−*^*Hmgn2*^*−/−*^ double knockout mice and control wild type mice were housed in an AAALAC-accredited facility for up to five per cage in individually ventilated caging on a 12:12 light:dark cycle with hardwood bedding chips (PJ Murphy, Montville, NJ) and nesting material (Nestlet, Ancare, Bellmore, NY). Autoclaved rodent chow (Teklad 7017/NIH-31, Envigo, Indianapolis, IL) and chlorinated city water (4–8 ppm) were available ad libitum. Room temperature and humidity were maintained at 72 ± 2 ^o^F (22.2 ± 1.1 °C) and 50% ± 20%, respectively. Health checks were performed twice daily assessing for attitude, activity level, and overall appearance of health. Cage changing occurred once every 2 weeks using tail-mediated transfer of individual animals. The IACUC-approved protocol upon which these animals were enrolled was monitored three times per week by investigator staff. Humane endpoints resulting in immediate euthanasia included hunched posture, rough hair coat, signs of dehydration (reduced skin turgor, sunken eyes), abnormal respiration (tachypnea, dyspnea, coughing), reduced or impaired mobility affecting the ability to obtain food or water, Pallor or cyanosis, hemorrhage or bleeding from any orifice, diarrhea, constipation or markedly reduced food intake; neurologic abnormalities (seizures, paralysis, circling, head tilt), impaired ability to urinate or defecate, visible jaundice, or loss of >15% normal body weight from pre-study baseline. CO_2_ euthanasia was performed according to the AVMA Guidelines for the Euthanasia of Animals (2020 Edition).

### Intermittent fasting and high-fat diet studies

8–10 weeks old mice were randomly grouped into the control or fasting group. The control group mice were allowed unrestricted access to food, while the fasting group had 24-h free access to food followed by 24 h of fasting. For the high-fat diet study, 8–10-week-old WT and DKO mice were fed with 60% high-fat diet (Bioserv, Cat# S3282) for 16 weeks. Body weights were measured every week, and food was replaced weekly.

### Cell culture

Mouse embryonic fibroblasts (MEFs) were prepared from E13.5 embryos and maintained in DMEM medium containing 10% FBS and 1% Pen Strep as described^47^. MEFs from WT and DKO within passage 3 were used for reprogramming. For adipocyte differentiation, MEFs were grown to confluence, and 2 days after confluence the cells were treated with differentiation medium (Insulin (0.5 μg/ml), dexamethasone (5 μM), IBMX (0.5 mM), T3 (1 nM), Rosiglitazone (1 μM)) for 8 days and then with maintenance medium (Insulin (0.5 μg/ml), T3 (1 nM)) for 4 days.

### Preadipocyte isolation and differentiation

The isolation procedure for primary white preadipocytes was adapted from Pydi et al.^48^. Subcutaneous inguinal fat (iWAT) from 8- to 12-week-old mice were minced and then digested at 37 °C for 1 h in phosphate-buffered saline (PBS) buffer containing collagenase 1 (3 mg/ml) (Sigma-Aldrich). The digested tissue suspension was filtered through a 100-μm cell strainer, followed by centrifugation at 1000 rpm for 5 min at room temperature. The cell pellet was resuspended in 5 mM EDTA (1 ml/mice) and sedimented at 1000 rpm for 5 min. in a Sorvall Legend RT + centrifuge. The cell pellets were resuspended in ACK lysis buffer (K.D. Medical, Columbia, MD), incubated for 5 min at RT, diluted 10X with PBS, and resedimented for 5 min. The cell pellets were resuspended in 10 ml DMEM media containing 10 FBS and 1% Pen strep and filtered through a 40-μm cell strainer and plated in 10 cm collagen-coated plates (Corning, NY). An equal number of cells were then counted and replated. Once the cells reached confluency, they were differentiated into brown-like adipocytes using differentiation media: (Insulin (0.5 μg/ml), dexamethasone (5 μM), IBMX (0.5 mM), T3 (1 nM), Rosiglitazone (1 μM)) for 4 days and maintained in maintenance medium (Insulin (0.5 μg/ml), T3 (1 nM)) for 4 days. For differentiating into mature white adipocytes, we treated preadipocytes with a white adipogenic media: (Insulin (0.5 μg/ml), dexamethasone (5 μM), IBMX (0.5 mM) for 4 days, and then in maintenance medium without T3: Insulin (0.5 μg/ml) for another 4 days.

### Plasmid DNA and siRNA

Plasmid DNA was transfected into cells using Lipofectamine 3000 reagent (Thermo Fisher Scientific, Cat# L3000015) according to the manufacturer’s protocol. SiRNAs were transfected using Lipofectamine RNA iMAX transfection reagent (Thermo Fisher Scientific, Cat# 13778150).

### RNA preparation and qRT-PCR assay

For total RNA isolation, cells or mouse tissues were collected in TRIzol reagent (Invitrogen, Cat# 15596026) and isolated as described in the manufacturer’s protocol. Then subjected to on-column DNaseI digestion and RNA purification using RNeasy Mini Kit (Qiagen). For RT-PCR, RNA was converted to cDNA using the SuperScript First-strand system (Thermo Fisher Scientific, Cat# 11904018). All qRT-PCR reactions were performed using the SYBR Green PCR master mix (Applied Biosystems, Cat# 4309155). Relative expression of genes was normalized to 18S rRNA. The relative fold change was calculated using the formula 2^−^^ΔΔCt^. A complete list of primer sequences is provided in Table S1.

### Western blot

Cells were lysed with CellLytic M buffer (Sigma, Cat# C2978) and freeze-thawed. Protein supernatant was collected by centrifuging the cell lysate at 10,000 rpm for 10 min at 4 °C and protein concentration was determined using nanodrop. 30–50 μg protein samples were resolved on 4–12% SDS–polyacrylamide gel (BioRad) and transferred onto PVDF membrane using iBlot Gel Transfer Stacks (Life Technologies). The following antibodies were used for Western blotting: anti-UCP1 (Abcam, Cat# ab10983) and anti-β-actin (Santacruz, Cat# sc-47778). Membranes were blocked with 5% skim milk and incubated overnight with primary antibodies followed by incubation with HRP-conjugated secondary antibodies. The blots were developed with Luminata Forte Western HRP substrate (Millipore). All the uncropped images of Western blot results related to Figs. 1q, 3c, e; 5g and Supplementary Fig. 8d and f are shown in Supplementary Fig. 9.

### Histology and immunofluorescence

Mouse tissues were fixed in a 10% formalin solution (Sigma-Aldrich, St. Louis, MO); paraffin sections were examined by staining with Hematoxylin and Eosin (H&E). For immunofluorescence, paraffin sections were deparaffinized by xylene solutions and rehydrated in serial ethanol solutions from high to low concentration. Antigen retrieval was done by heating sections in 10 mM citric acid buffer (pH 6.0) in a microwave oven for 10 min and followed by blocking buffer (5% normal goat serum in 1X PBS) treatment for one hour at room temperature. The primary antibody for UCP1 (Abcam, Cat# ab10983) was diluted into antibody dilution buffer (1% BSA in 1X PBS). Nuclear staining was done with DAPI (Thermo Fisher Scientific). All the immunofluorescence images were taken with a Keyence BZ-X710 microscope.

### RNA-seq

Total RNA was prepared as described in RNA preparation. Strand-specific libraries were generated using the TruSeq Stranded Total RNA Library Prep Kit (Illumina). cDNA libraries were paired-end sequenced (50 bp) on an Illumina HiSeq 3000. Reads were aligned to the mouse genome (NCBI37/mm10) with STAR. Downstream analysis was conducted with EdgeR R package.

### Single-cell RNA (scRNA) sequence analysis

Single-cell suspensions were prepared from WT and DKO cell cultures in biological duplicate at Day 0 and Day 6 of in vitro browning. Single-cell suspensions were washed twice with PBS with 0.04% BSA before resuspension and counting. The sample concentration and viability were assessed using the LunaFL fluorescent cell counter (Logos Biosystems) with acridine orange and propidium iodide staining. Individual single-cell suspension samples were diluted and loaded onto separate captures lanes of the Chromium Connect platform (10x Genomics) using 3’ v3.1 Single Cell RNA-Seq chemistry reagents (10x Genomics) with a target recovery of 6000 cells per capture lane. Each biological replicate condition was partitioned and barcoded on separate capture lanes. Cell partitioning, reverse transcription, cDNA amplification, and all steps of library preparation were performed according to the manufacturer’s specifications on the Chromium Connect instrument. Single-cell libraries were quantified, pooled, and sequenced on the NextSeq 2000 instrument (Illumina) to a target read depth of ~50,000 reads per cell on average. Sequencing data were demultiplexed and mapped to the mouse genome (mm10: refdata-gex-mm10-2020-A) using Cell Ranger (10X Genomics, version 6.0.0) to generate a single cell gene expression matrix. The filtered gene expression matrix from aggregated and read-depth normalized data generated by Cell Ranger was imported to Seurat v4 (https://satijalab.org/seurat/^49^). After importing and filtering cells that expressed fewer than 500 genes and more than 10% mitochondrial gene expression, the dataset was log normalized by a scale factor of 10,000 and scaled by regressing out the variation associated with mitochondrial reads, followed by principal component analysis using the variable features. The clustering step was performed, taking in the first 10 PCs, with both the low resolution of 0.1 and high resolution of 0.6, generating 7 and 14 clusters, respectively. The steps of scaling to clustering were repeated after removing unwanted cells (immune cells, glial, and endothelial cells) and 4 clusters were generated with a low resolution of 0.05 while 12 clusters were produced with a high resolution of 0.6. Differential expression was calculated using every cluster against the remaining clusters using the FindAllMarkers function of the Seurat package using MAST method^50^ with min pct 0.1 and log fold change threshold 0.25.

Cell trajectory and pseudo-time analysis were performed using the Monocle R package (v2.22.^51^), using the top 100 gene loadings from the principal component deemed best to represent brown adipocyte differentiation to order the cells. We have chosen the root based on the adipocyte gene expression pattern. The preadipocyte genes have higher expression in the root and differentiated beige adipocyte markers show higher expression toward the left branch. Before creating the percentages of cells from each sample within each pseudo-time trajectory state, we calculated and applied a scaling factor to ensure no sample representation bias.

### ChIP seq

WT or DKO preadipocytes and adipocyte cells were crosslinked with 1% formaldehyde (v/v) for 10 min at room temperature on a rocking platform, followed by quenching with 125 mM glycine. Crosslinked cells were washed twice with ice-cold phosphate-buffered saline, and ~1 × 10^7^ cells were incubated in 1 ml chromatin prep buffer containing 1 μl proteinase inhibitor cocktail (PIC) and 1 μl of 100 mM PMSF (Active Motif) for 10 min on ice followed by centrifuging for 3 min at 1250×*g* at 4 °C. The pellets were resuspended in 250 μl ChIP buffer with 2.5 μl PIC and 2.5 μl 100 mM PMSF and sonicated for 30 cycles with Bioruptor (30 s on/30 s off). Aliquots of 25 μl of sonicated chromatin were used for input DNA preparation. 5 μg of specific antibodies were then added to the rest of the chromatin samples and incubated overnight at 4 °C with rotation. Following incubation, 30 μl of protein G agarose beads (Active Motif) was then added to each reaction and the mixtures were further incubated for 3 h at 4 °C. The beads were collected by centrifugation and washed five times with Wash Buffer AM1 (Active Motif). ChIP DNA was eluted in 100 μl Elution Buffer AM4 (Active buffer). Crosslinks were reversed at 65 °C overnight in the presence of 3 μl of 10% SDS and 5 μl of proteinase K (20 mg/ml). The DNA samples were eluted in 21 μl of elution buffer using a MiniElute kit (Qiagen). The ChIP-seq library was prepared following the manufacturer’s instructions (Illumina). Briefly, immunoprecipitated and input DNA was blunt-ended, ligated to adapters, amplified with PCR, and size selected. The ChIP templates were sequenced at 75 bp single read length with Illumina NextSeq 500 system by the NIH CCR-sequencing facility. Sequence reads were aligned to the mouse genome data (NCBI37/mm10). The antibodies used in chromatin immunoprecipitation include Rabbit polyclonal to H3K27ac (Abcam, ab4729).

### ATAC seq

ATAC-Seq libraries were prepared from 50,000 cells using the Nextera Tn5 Transposase and DNA library preparation kit (Illumina) as described previously^23^. Libraries were paired-end sequenced (50 bp) on a NextSeq 500. Reads were mapped to the mouse genome (NCBI37/mm10) using Bowtie2. Reads were removed from the subsequent analysis if they were duplicated, mapped to the mitochondrial genome, or aligned to unmapped contiguous sequences. Peak calling was performed using MACS2 using default parameters. The reads were converted to reads per thousand base pairs peak per million mapped reads (RPKM) by dividing by the total number of reads per sample.

### Indirect calorimetry

Total energy expenditure (TEE), oxygen consumption rate, food intake (floor feeder), and physical activity (infrared beam break as total activity, 0.5-inch spacing) were measured by indirect calorimetry (CLAMS using Oxymax software v5.52, Columbus Instruments, Columbus, OH) for 3 days at 22 °C. Mice were housed individually with ad libitum access to food and water in chambers without bedding or nesting material (2.5 L volume, flow rate 0.5 L/min, sampling flow 0.4 L/min, settle time 55 s, measure time 5 s, each chamber sampled every 13 min, giving 5 sampling cycles per 65 min interval). The food intake and physical activity were measured at 13-min intervals for several weeks. All 12 calorimetry chambers were housed in a single temperature-controlled environmental chamber^52,53^.

### Body composition analysis

Body composition (lean/fat mass) of WT and DKO mice was measured by time domain Echo MRI 3-in-1 (Echo Medical System, Houston, TX).

### Estimating energy expenditure by energy balance technique

Food intake and body composition were measured weekly over a period of 7 weeks in mice housed individually in their home cages and fed a chow diet (7022 NIH-07 diets, Envigo Inc., Indianapolis, IN). Energy expenditure was calculated from the metabolizable caloric intake, corrected for the change in caloric content of the mouse (from the change in body composition over the measurement interval)^53^.

### Statistics

Results are expressed as the mean ± SD unless otherwise mentioned. Two-tailed *t*-tests were performed to obtain *p*-values. Statistical significance was established at *p < 0.05.

### Reporting summary

Further information on research design is available in the Nature Portfolio Reporting Summary linked to this article.

## Supplementary information

Supplementary Information

Description of Additional Supplementary Files

Supplementary Data 1

Supplementary Data 2

Supplementary Data 3

Supplementary Data 4

Supplementary Data 5

Supplementary Data 6

Supplementary Data 7

Supplementary Data 8

Reporting Summary

## Data Availability

The RNA-seq, ChIP-seq, ATAC-seq, and scRNAseq data reported in this paper are available with the accession numbers: GSE193338, GSE193333, GSE193332, and GSE193462. Source data are provided with this paper, and uncropped gel/blot images are included at the end of the Supplementary Information file as Supplementary Fig. S9. All other data generated or analyzed during this study are included in this published article (and its supplementary information files). Source data are provided with this paper.

## Source data

Source Data
