# Supplementary material for: Epigenetic regulation of white adipose tissue plasticity and energy metabolism by nucleosome binding HMGN proteins
Source: Nat Commun. 2022 Nov 26;13:7303. doi: 10.1038/s41467-022-34964-5 (PMC9701217; doi:10.1038/s41467-022-34964-5)
Supplement: Supplementary file 1 — Supplementary Information [file 41467_2022_34964_MOESM1_ESM.pdf]

Supplementary material for

Epigenetic Regulation of White Adipose Tissue Plasticity and Energy Metabolism By Nucleosome Binding HMGN Proteins

Ravikanth Nanduri[1*], Takashi Furusawa[1], Alexei Lobanov[2], Bing He[1], Cen Xie[3], Kimia Dadkhah[3], Michael C. Kelly[3], Oksana Gavrilova[4], Frank J. Gonzalez[5] and Michael Bustin[1*]

[1]Protein Section, Laboratory of Metabolism, Center for Cancer Research, National Cancer Institute, National Institutes of Health, Bethesda, MD, 20892, USA. [2]CCR Collaborative Bioinformatics Resource, Center for Cancer Research, National Cancer Institute, National Institutes of Health, Bethesda, MD, USA. [3]Nucleic Acid Section, Laboratory of Metabolism, Center for Cancer Research, National Cancer Institute, National Institutes of Health, Bethesda, MD, 20892, USA [4]CCR Single Analysis Facility, Cancer Research Technology Program, Frederick National Laboratory for Cancer Research, Bethesda, MD 20892, USA,[5]Mouse Metabolism Core Laboratory, National Institute of Diabetes and Digestive and Kidney Diseases, National Institutes of Health, Bethesda, MD 20892, USA.

*Corresponding author: Bldg. 37 Room 3122, 37 Convent Drive, Bethesda, MD 20892, US

email: bustinm@mail.nih.gov MB: https://orcid.org/0000-0002-5147-7242
ravikanth.nanduri@nih.gov RN: https://orcid.org/0000-0002-0031-2333

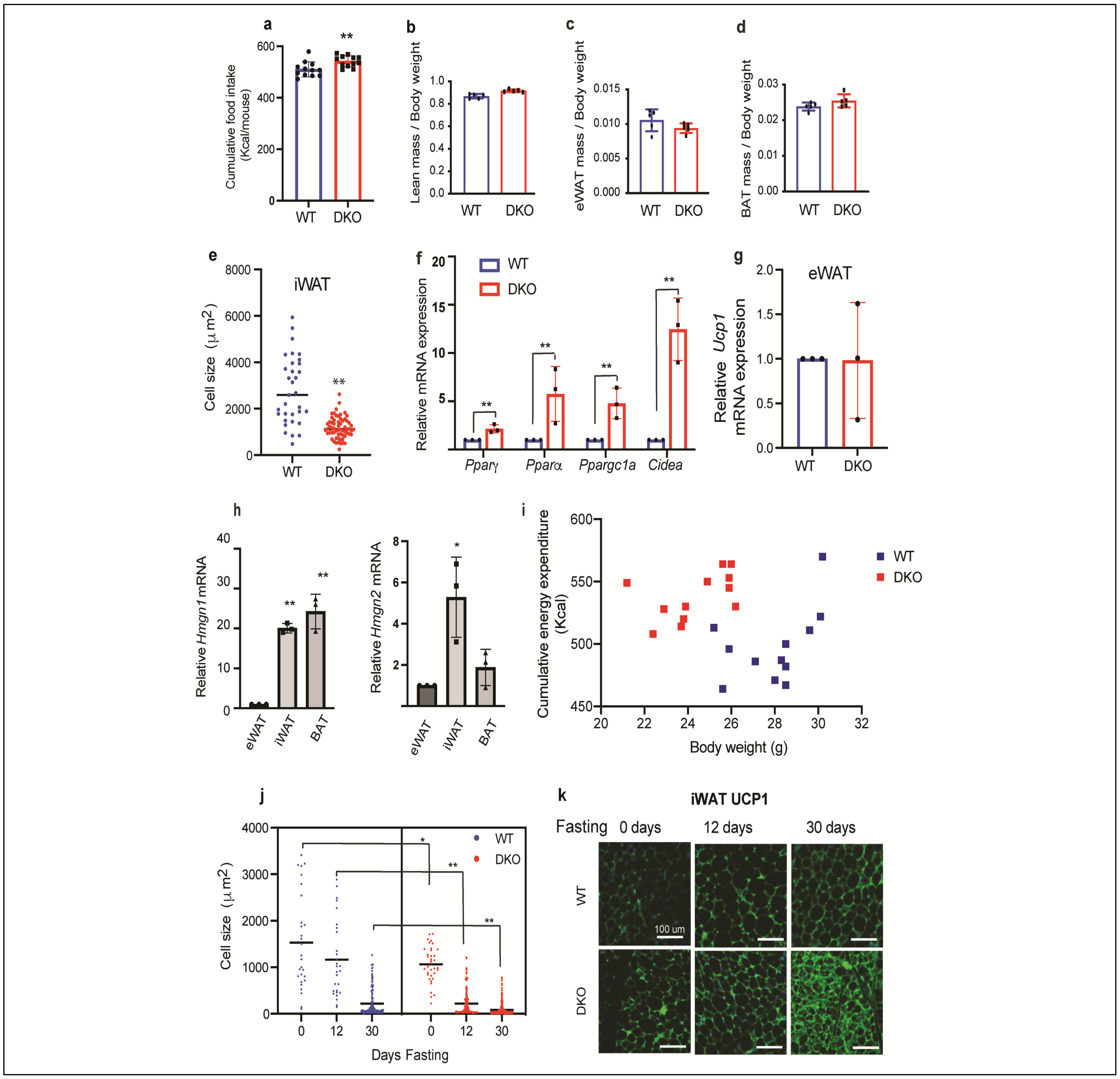

Fig. S1. Enhanced white adipose tissue browning in HMGN DKO mice.

a) Elevated cumulative food intake in DKO mice measured for 7 weeks. (n=12 for each group). **b**) Lean mass of 25 weeks old WT and DKO mice. Tissue mass was normalized to body weight (n = 5 for each group). **c)** eWAT mass of 25 weeks old WT and DKO mice. Tissue mass normalized to body weight (n = 5 for each group). **d)** BAT weight of 25 weeks old WT and DKO mice. Tissue mass normalized to body weight (n = 5 for each group). **e)** Decreased adipocytes size of DKO iWAT cells. Size H&E stained cell determined by BZ-X analyzer (Keyence). **f)** Quantitative RT-PCR analysis of transcript levels in iWAT of WT and DKO mice (n = 3 for each group). **g)** Quantitative RT-PCR analysis of *Ucp1* expression in eWAT from WT and DKO mice (n = 3 for each group). **h)** Quantitative RT-PCR analysis of *Hmgn1* and Hmgn2 expression in iWAT, eWAT and BAT from WT and DKO mice (n = 3 for each group). **i)** Cumulative energy expenditure during 7 weeks (mice aging from 11-17 weeks) vs body weight measured in 17 weeks old WT and DKO mice. n=12 per group. **j)** Size of H&E stained WT and DKO iWAT adipocytes during fasting. **k)** Immunostaining of UCP1 in iWAT from fasting WT and DKO mice. Data are the mean ± S.D. A two-tailed t-test was used in 1a, f, h, j; *, p values < 0.05. For 1k, data are representative of two independent experiments with similar results.

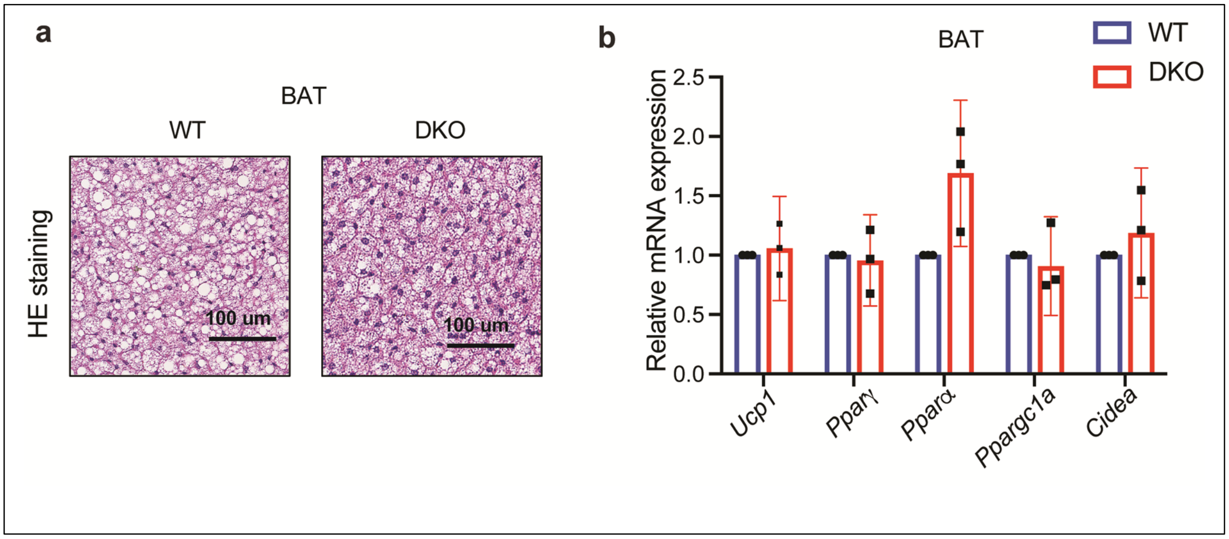

Fig. S2. Loss of HMGN does not alter adipocyte browning in BAT. a) H&E staining of BAT cells from WT and DKO mice. **b**) Quantitative RT-PCR analysis of genes associated with adipocyte browning in BAT from WT and DKO mice. Data are the mean ± S.D; n=3 for each group.

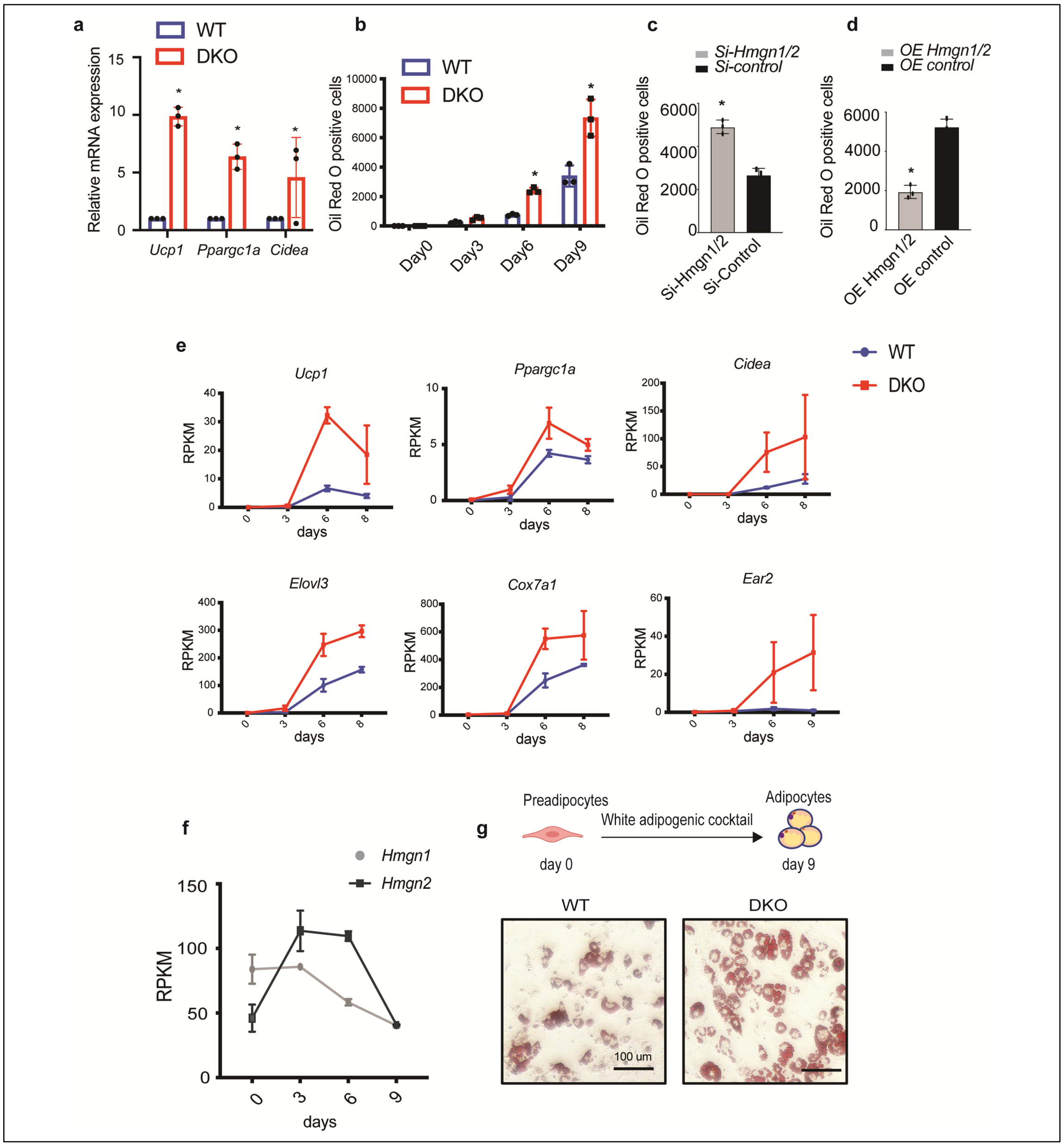

Fig. S3. Enhanced differentiation efficiency of DKO white preadipocytes into brown-like adipocytes. a) Quantitative RT-PCR analysis of brown adipocyte-specific genes *Ucp1*, *Pgc1a* and *Cidea in vitro* differentiated beige adipocytes from WT and DKO white preadipocytes. **b)** Quantification of Oil Red O positive cells in fig 3B, represented as bar histograms. **c)** Quantification of Oil Red O positive cells in fig 3D, represented as bar histograms. **d)** Quantification of Oil Red O positive cells of fig 3F, represented as bar histograms. **e)** Expression levels of representative brown adipocyte-specific genes such as *Ucp1*, *Pgc1a*, *Cidea*, *Elvol3*, *Cox7a1,* and *Ear2* during *in vitro* differentiation of WT and DKO white preadipocytes into beige adipocytes. **f)** HMGN transcript levels during preadipocyte browning. **g)** Oil Red-O staining of *in vitro* differentiated adipocytes from WT and DKO white preadipocytes using a white adipogenic cocktail. Data are the mean ± S.D; n=3 for each group. A two-tailed t-test was used in 1a-d; *, $p < 0.05$.

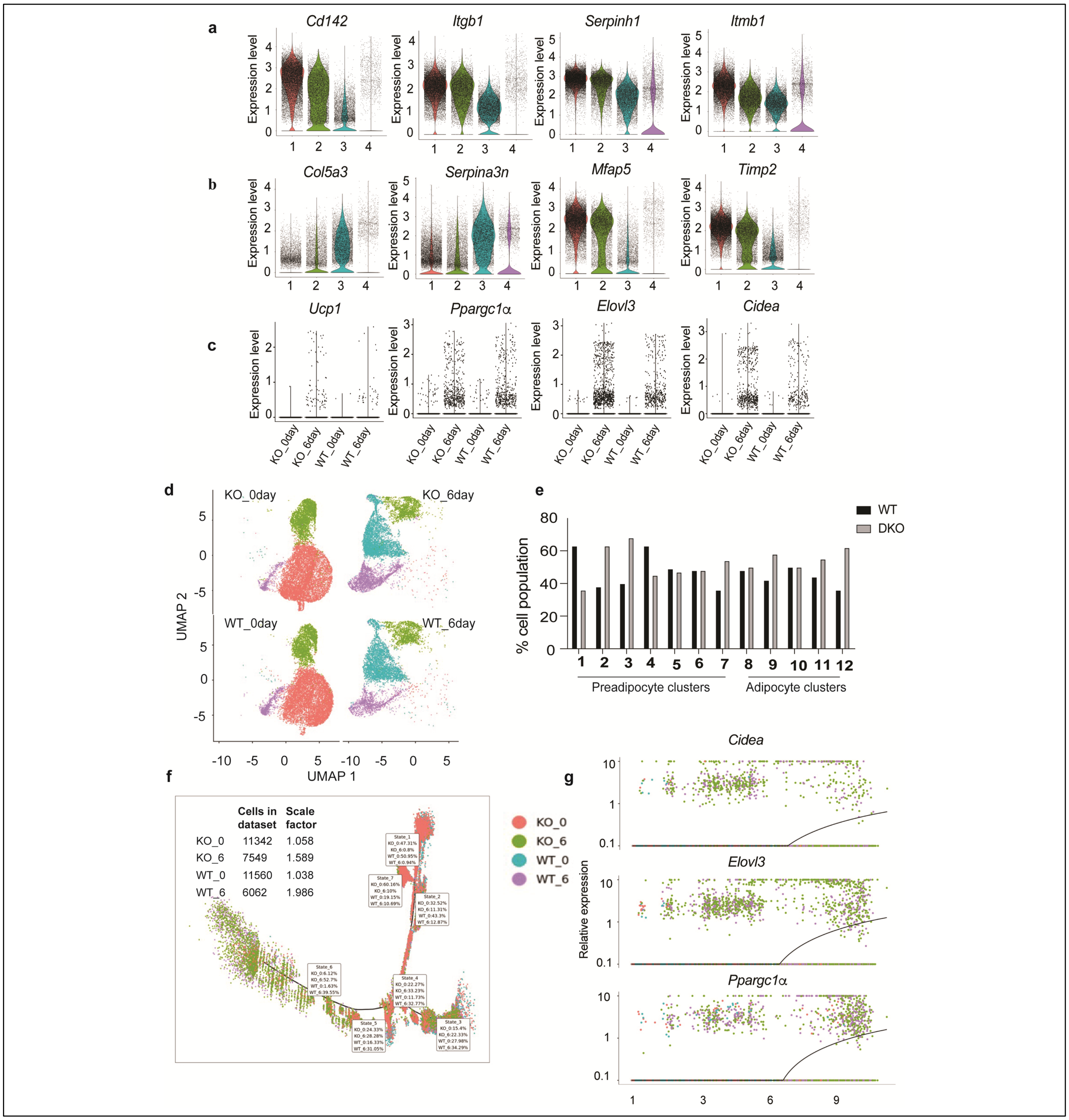

Fig. S4. Aggregate Single-cell RNA seq analysis at day 0 and 6 during browning of white preadipocytes. a) Expression of white preadipocyte marker genes in various clusters shown in Fig 4B. **b)** Expression of differentiating adipocyte marker genes in various clusters shown in Fig 4B. **c)** Expression of brown adipocyte marker genes in various clusters shown in Fig 4B split by genotype. **d)** UMAP plots split by genotype showing various clusters of aggregate scRNAseq analysis at day 0 and 6 during browning of white preadipocytes. Data from duplicate samples of either WT or DKO cells. **e)** Bar graph showing WT and DKO % cell populations in the clusters identified in panel D. n=2 per group. Shown are the average values of two biological replicates. **f)** Trajectory analysis of aggregated scRNA-seq data. Trajectory starts from top and goes down, forming two primary branches to the bottom left and bottom right in this plot. **g)** Expression of brown adipocyte marker genes later in the pseudo time of trajectory analysis.

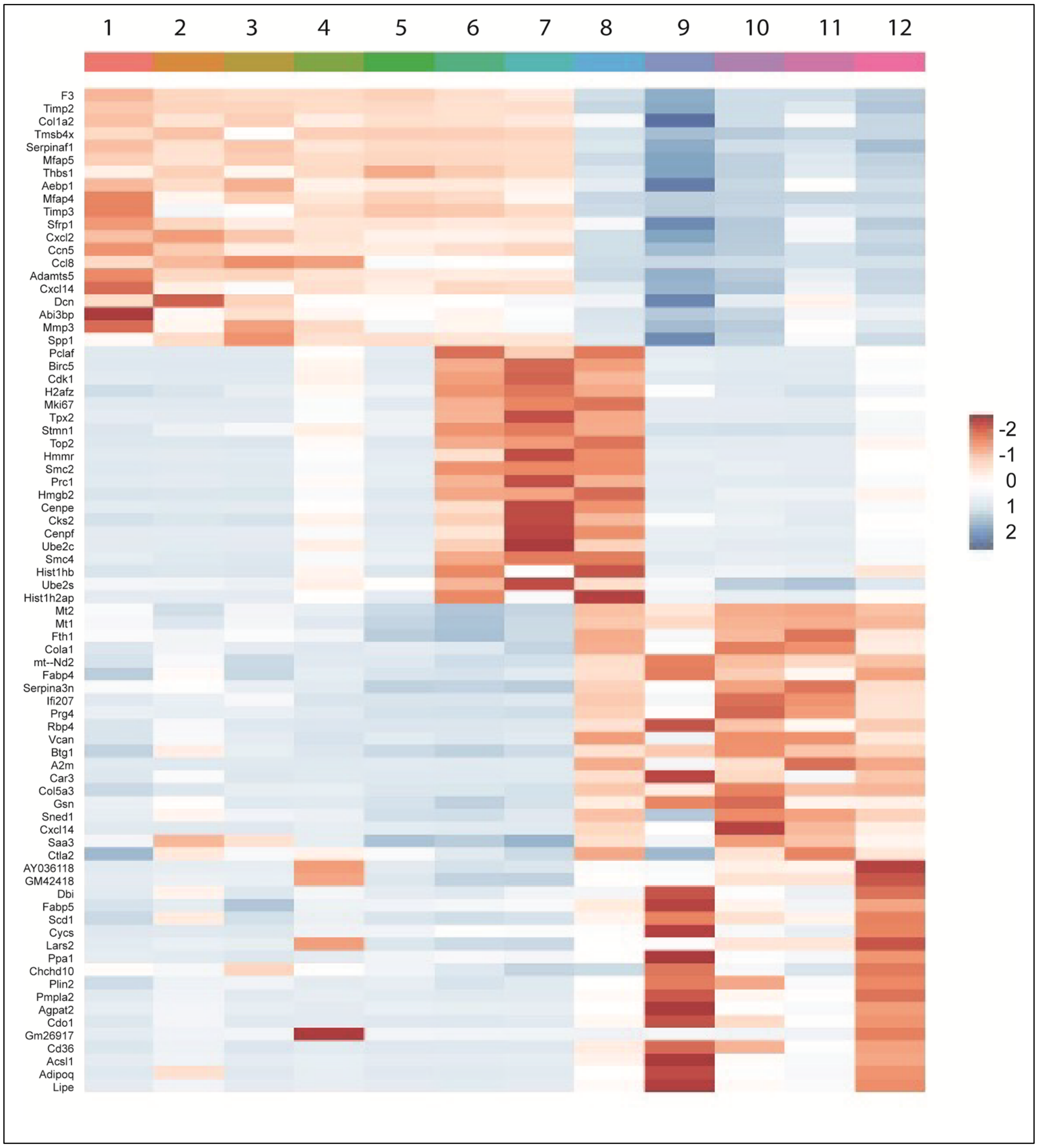

Fig. S5. **Heatmap showing major genes altered in different clusters represented in Fig 4E.**

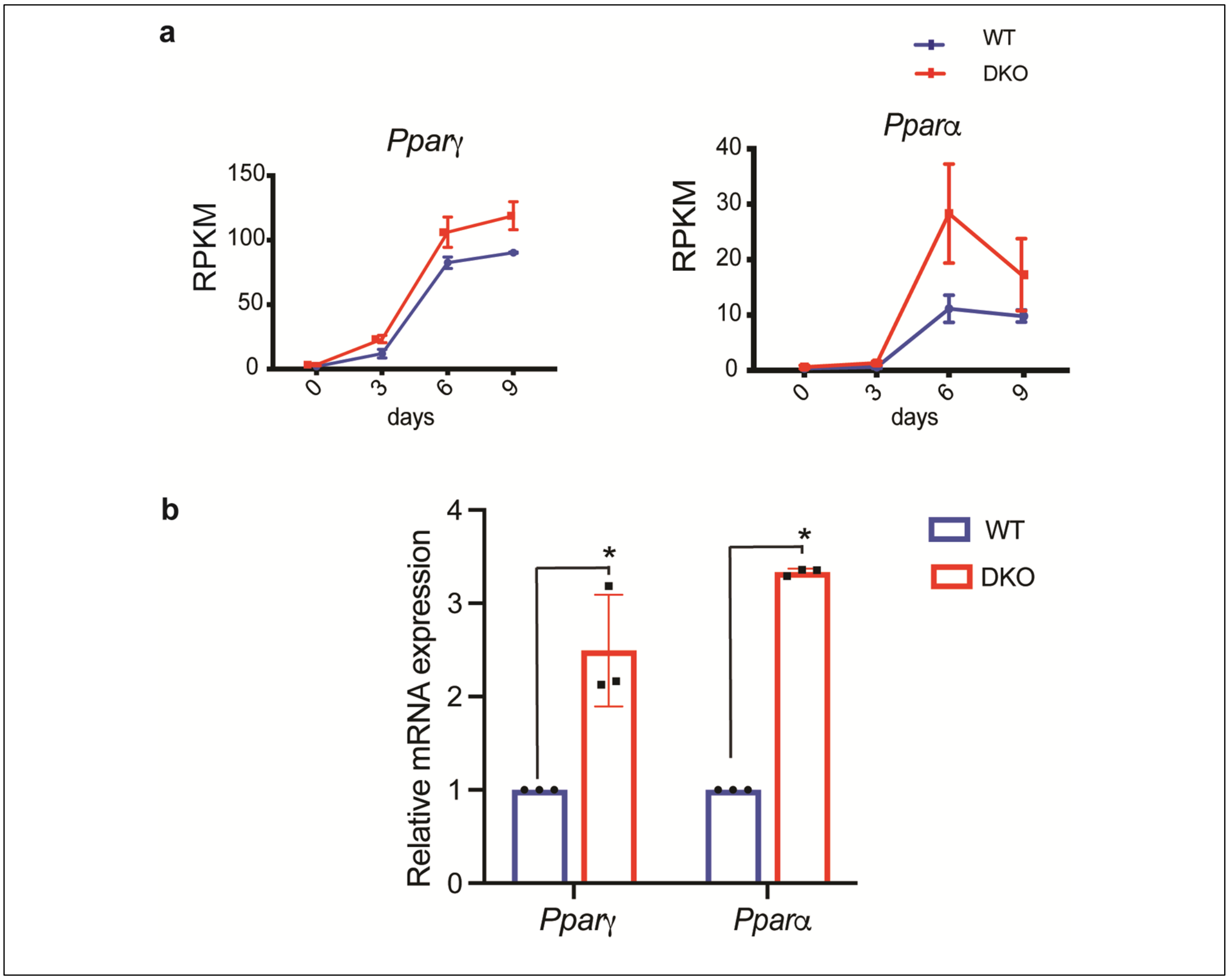

Fig. S6. Expression of Pparγ and Pparα during browning of WT and DKO white preadipocytes. a) Expression levels of *Pparγ* and *Pparα* during *in vitro* differentiation of WT and DKO white preadipocytes into beige adipocytes, determined by RNA seq. **b)** Expression levels of *Pparγ* and *Pparα* mRNA at day 9 of differentiation of WT and DKO white preadipocytes into beige adipocytes, determined by qRT-PCR. Data are the mean ± S.D; n=3 for each group. *, $p < 0.05$.

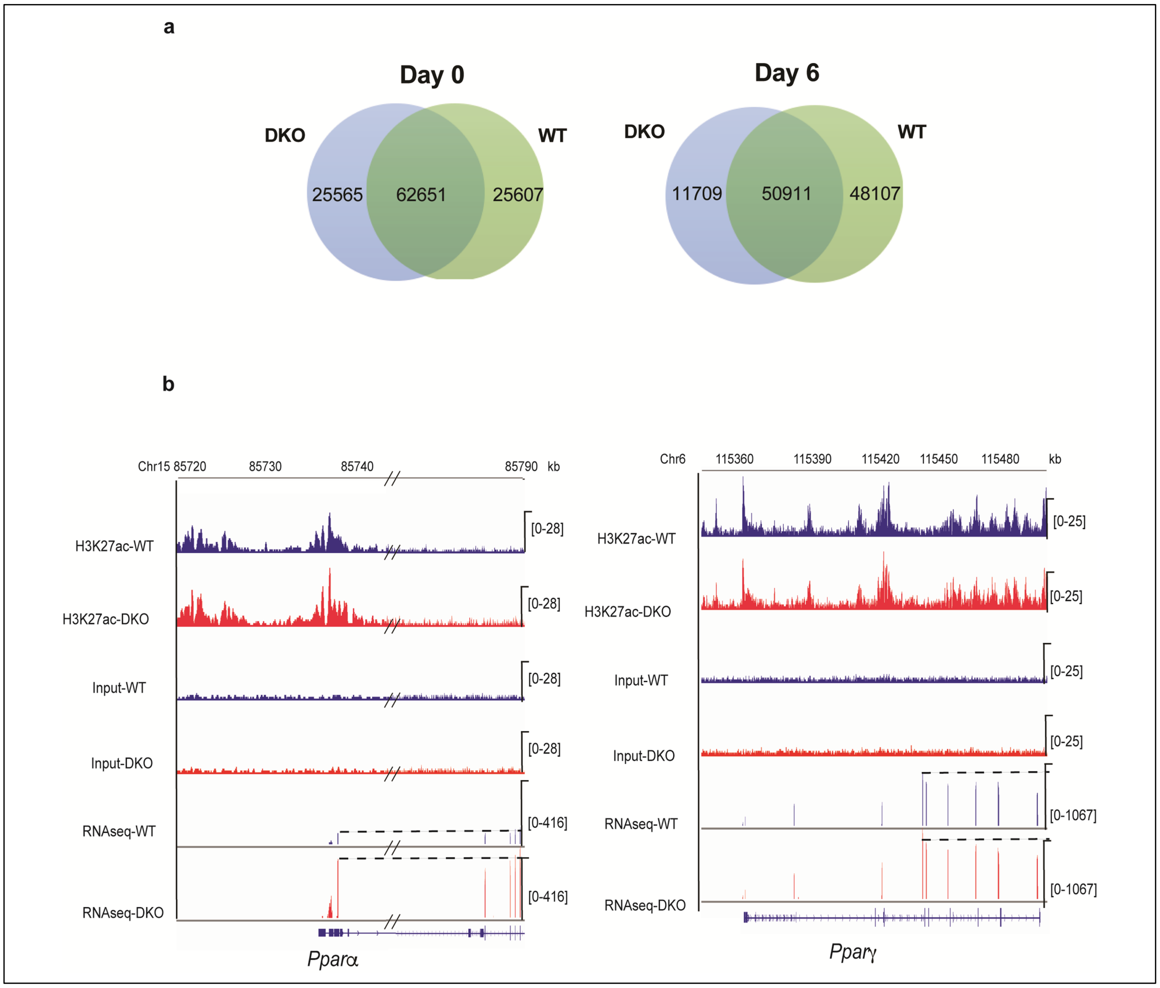

Fig. S7. Loss of HMGN alters H3K27ac levels in adipocytes. a) Venn diagram showing number of common and unique H3K27ac sites in WT and DKO at Day 0 and day 6 of preadipocyte differentiation. Data are from 3 biological replicates. **b)** IGV screenshots showing H3K27ac and transcript levels of *Pparγ* and *Pparα* at day 6 of preadipocyte differentiation.

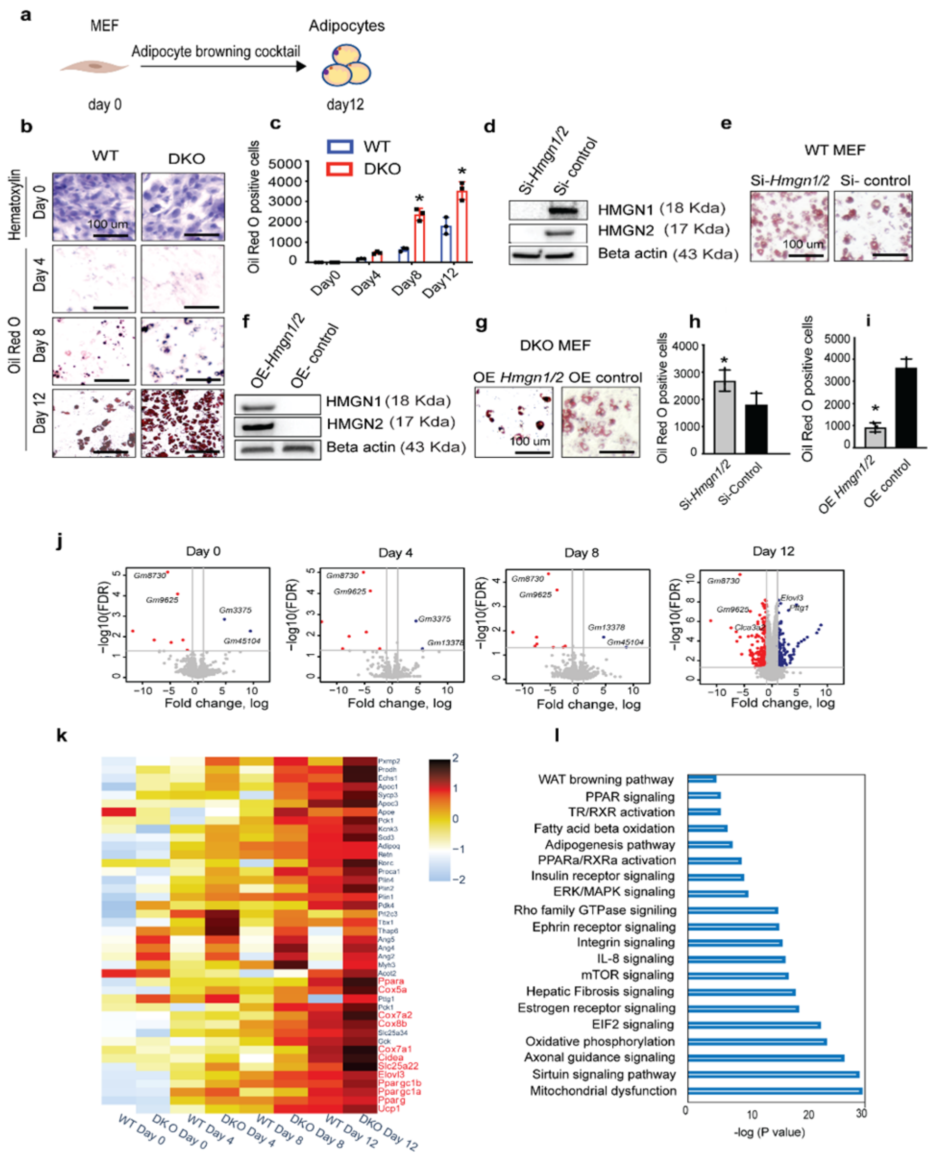

Fig. S8: Enhanced differentiation efficiency of DKO mouse embryonic fibroblasts (MEFs) into brown-like adipocytes. a) Scheme of *in vitro* differentiation of MEFs into mature adipocytes. Created with Biorender.com. **b)** Oil Red O staining of WT and DKO adipocytes differentiated from MEFs. 0 day were stained with hematoxylin, and 4th, 8th and 12th day were stained with Oil Red O. **c)** Quantification of Oil Red O positive cells in fig S8b, represented as bar histograms. Data are the mean ± S.D; *, $p < 0.05$. **d)** Western blot analysis of HMGN1 and HMGN2 expression in WT MEFs transfected with *Hmgn1* and *Hmgn2* siRNA or Scrambled siRNA. **e)** Oil Red O staining of *in vitro* differentiated WT MEFs transfected with *Hmgn1* and *Hmgn2* siRNA or Scrambled siRNA. **f)** Western blot analysis of HMGN1 and HMGN2 expression in DKO MEFs transfected with *Hmgn1* and *Hmgn2* expression plasmids or control plasmids. **g)** Oil Red O staining of *in vitro* differentiated DKO MEFs transfected with *Hmgn1* and *Hmgn2* expression plasmids or control plasmids. **h)** Quantification of Oil Red O positive cells in fig S8e, represented as bar histograms. Data are the mean ± S.D; n=3 for each group. *, $p < 0.05$. **i)** Quantification of Oil Red O positive cells of fig S8g, represented as bar histograms. Data are the mean ± S.D; n=3 for each group. *, $p < 0.05$. **j)** Volcano plots showing differential gene expression (fold change ≥ 1.5; FDR < 0.05) between WT and DKO adipocytes differentiated from MEFs. A list of all the differentially expressed genes are shown in supplementary data 8. **k)** Heatmap showing enhanced upregulation of genes associated with white adipocyte browning during DKO MEFs differentiation into adipocytes, compared to WT. **l)** IPA analysis of preferentially regulated pathways in DKO adipocytes during MEFs differentiation at day 12. Data are from 3 biological replicates.

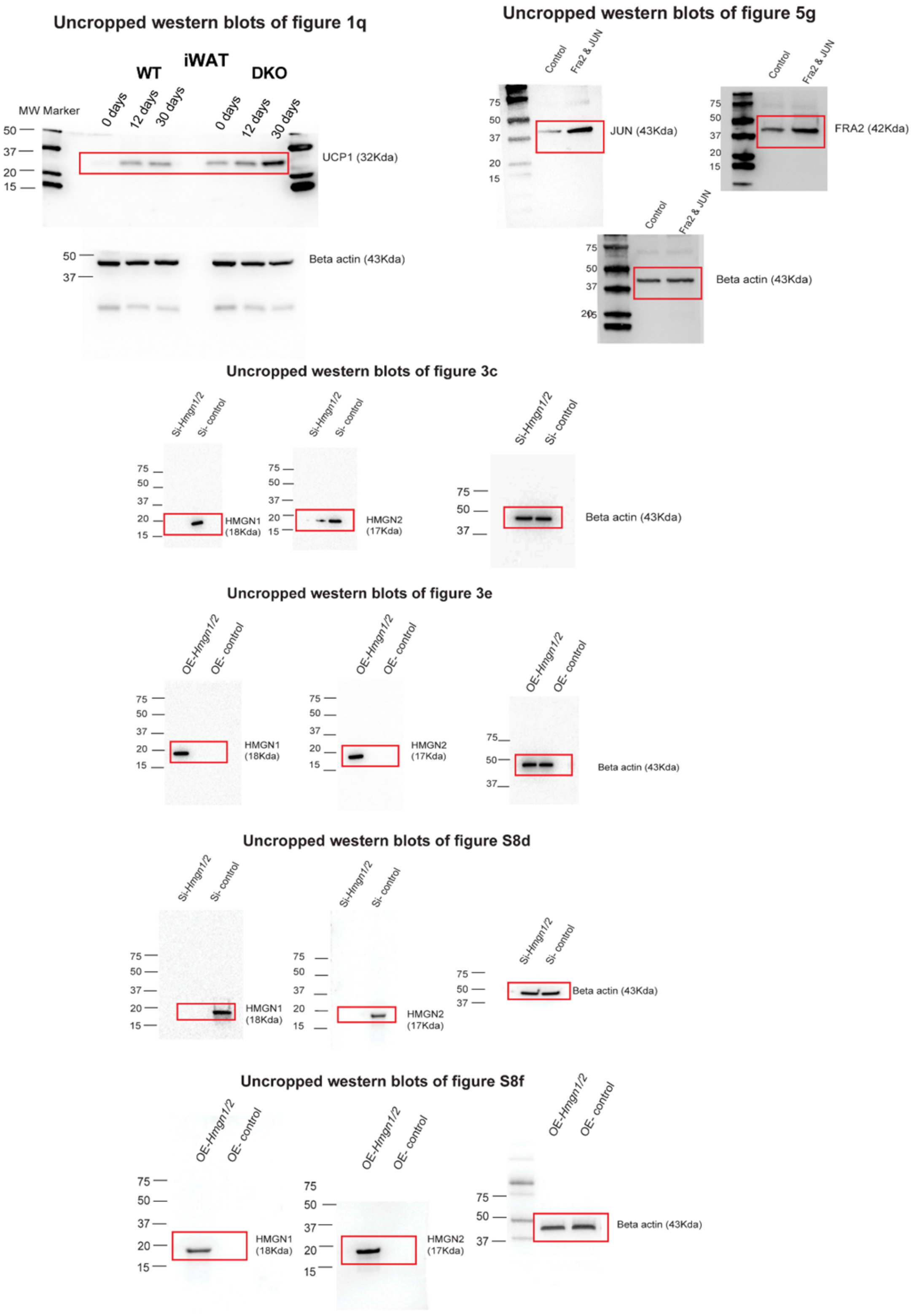

Fig. S9: Images of uncropped western blots shown in figure 1, 3, and S8.

Table S1 Primers used for quantitative real-time PCR

Primer name	Forward	Reverse
mCoxII mtDNA	GCCGACTAAATCAAGCAACA	CAATGGGCATAAAGCTATGG
mß-Globin gDNA	GAAGCGATTCTAGGGAGCAG	GGAGCAGCGATTCTGAGTAGA
Ucp1	ACTGCCACACCTCCAGTCATT	CTTTGCCTCACTCAGGATTGG
Cidea	TGCTCTTCTGTATCGCCCAGT	GCCGTGTTAAGGAATCTGCTG
18s	CGGCTACCACATCCAAGGAA	GCTGGAATTACCGCGGCT
Ppar α	TGAGGAAGCCGTTCTGTGAC	GTTTAGAAGGCCAGGCCGAT
Ppar γ	GCACTGCCTATGAGCACTTCA	GGTCTTCCATCACGGAGAGG
Ppargc1 α	AGCCGTGACCACTGACAACGAG	GCTGCATGGTTCTGAGTGCTAAG
PPAR α Promoter	TGGCATAGCACACATTTCGT	GCTTTGCTTGTGTCGTCCTT
PPAR γ Promoter	TACCAAGTCTTGCCAAAGCA	GGGAGAGGTGGGAATAAACA
