# Supplementary material for: Epigenetic regulation of white adipose tissue plasticity and energy metabolism by nucleosome binding HMGN proteins
Source: Nat Commun. 2022 Nov 26;13:7303. doi: 10.1038/s41467-022-34964-5 (PMC9701217; doi:10.1038/s41467-022-34964-5)
Supplement: Supplementary file 2 — Description of Additional Supplementary Files [file 41467_2022_34964_MOESM2_ESM.pdf]

Description of Additional Supplementary Files

File Name: Supplementary Data 1
Description: Cumulative food intake and energy expenditure (kcal) from week 11-17

File Name: Supplementary Data 2
Description: Differential expressed genes from RNA seq of Preadipocyte browning

File Name: Supplementary Data 3
Description: List of Differentially expressed genes in different clusters from low resolution cluster analysis

File Name: Supplementary Data 4
Description: ATAC nearest genes.xlsx

File Name: Supplementary Data 5
Description: List of transcription factor binding motifs showing downregulated ATAC sensitivity in DKO at day 0. Motifs identified by HOMER motif analysis.

File Name: Supplementary Data 6
Description: H3K27ac MA plot annotations

File Name: Supplementary Data 7
Description: List of white and brown adipocyte specific enhancers showing altered levels of H3K27ac and transcription during DKO and WT white preadipocytes browning.

File Name: Supplementary Data 8
Description: Differential expression of genes from RNA seq of MEF browning
