# Supplementary material for: Epigenetic regulation of white adipose tissue plasticity and energy metabolism by nucleosome binding HMGN proteins
Source: Nat Commun. 2022 Nov 26;13:7303. doi: 10.1038/s41467-022-34964-5 (PMC9701217; doi:10.1038/s41467-022-34964-5)
Supplement: Supplementary file 11 — Reporting Summary [file 41467_2022_34964_MOESM11_ESM.pdf]

nature portfolio

Corresponding author(s): Michael Bustin

Last updated by author(s): Oct 24, 2022

Reporting Summary

Nature Portfolio wishes to improve the reproducibility of the work that we publish. This form provides structure for consistency and transparency in reporting. For further information on Nature Portfolio policies, see our Editorial Policies and the Editorial Policy Checklist.

Statistics

For all statistical analyses, confirm that the following items are present in the figure legend, table legend, main text, or Methods section.

n/a	Confirmed	
☐	☒	The exact sample size (*n*) for each experimental group/condition, given as a discrete number and unit of measurement
☐	☒	A statement on whether measurements were taken from distinct samples or whether the same sample was measured repeatedly
☐	☒	The statistical test(s) used AND whether they are one- or two-sided<br>*Only common tests should be described solely by name; describe more complex techniques in the Methods section.*
☐	☒	A description of all covariates tested
☐	☒	A description of any assumptions or corrections, such as tests of normality and adjustment for multiple comparisons
☐	☒	A full description of the statistical parameters including central tendency (e.g. means) or other basic estimates (e.g. regression coefficient) AND variation (e.g. standard deviation) or associated estimates of uncertainty (e.g. confidence intervals)
☐	☒	For null hypothesis testing, the test statistic (e.g. *F*, *t*, *r*) with confidence intervals, effect sizes, degrees of freedom and *P* value noted<br>*Give P values as exact values whenever suitable.*
☐	☒	For Bayesian analysis, information on the choice of priors and Markov chain Monte Carlo settings
☐	☒	For hierarchical and complex designs, identification of the appropriate level for tests and full reporting of outcomes
☒	☐	Estimates of effect sizes (e.g. Cohen's *d*, Pearson's *r*), indicating how they were calculated

Our web collection on statistics for biologists contains articles on many of the points above.

Software and code

Policy information about availability of computer code

Data collection	ImageJ Schneider et al., 2012 https://imagej.nih.gov/ij/ (version 1.51)<br>indirect calorimetry (CLAMS using Oxymax software v5.52, Columbus<br>482 Instruments, Columbus, OH)<br>time domain Echo MRI 3-in-1 (Echo Medical System, Houston, TX).<br>Keyence BZ- X710 microscope.
Data analysis	Genome View IGV 2.6.2 http://software.broadinstitute.org/software/igv/home<br>RNA seq and ChIP Seq: Data were processed with CCBR Pipeliner (https://github.com/CCBR/Pipeliner)<br>RNA seq and ChIP seq: raw reads were trimmed with Cutadapt v1.18 (doi:10.14806/ej.17.1.200)<br>ChIP seq: Burrow-Wheeler Aligner BWA ver 0.7.17 (arXiv:1303.3997v2) was used to align reads to mm10 mouse genome<br>RNA seq: reads were mapped to mm10 mouse genome using STAR v2.7.0f (doi:10.1093/bioinformatics/bts635) in 2-pass mode.<br>CHIP seq quality control: FastQC (http://www.bioinformatics.babraham.ac.uk), PhantomPeakQualTools (doi: 10.1101/gr.136184.111), DeepTools v3.0.1 (doi:10.1093/nar/gkw257), Preseq v2.0.3 (doi: 10.1007/s40484-015-0049-7), FastqScreen v0.9.3 (doi:10.12688/f1000research.15931.2) and Kraken (doi:10.1186/gb-2014-15-3-r46) packages .<br>ChIP seq peak calling: MACS2 (doi: 10.1186/gb-2008-9-9-r137) and SICER v1.1 (doi: 10.1007/978-1-4939-0512-6_5)<br>ChIP seq differential binding was assessed with DiffBind (DOI: 10.18129/B9.bioc.DiffBind).<br>RNA-seq : gene-level expression was quantified using RSEM v1.3.0 (doi:10.1093/bioinformatics/bts635), with counts normalized to library size as counts-per-million.<br>RNA seq: limma-voom v3.34.5 (doi:10.1186/1471-2105-12-323) was utilized for quantile normalization and differential expression.<br>ATAC seq: Reads were mapped to the mouse genome (NCBI37/mm10) using Bowtie2<br>ATAC seq: Peak calling was performed using MACS2 using default parameters. The reads were converted to reads per thousand base pairs

peak per million mapped reads (RPKM) by dividing by the total number of reads per sample.
ScRNA seq: Sequencing data were demultiplexed and mapped to the mouse genome (mm10: refdata-gex-mm10-2020-A) using cellranger (10X Genomics, version 6.0.0) to generate a single cell gene expression matrix.
ScRNA seq: filtered gene expression matrix from aggregated and read depth normalized data generated by cellranger were imported to Seurat v4 (https://satijalab.org/seurat/
ScRNA seq: Differential expression was calculated using every cluster against the remaining clusters using the FindAllMarkers function of the Seurat package using MAST method
ScRNA seq: Cell trajectory and pseudo-time analysis was performed using the Monocle R package (v2.22)

For manuscripts utilizing custom algorithms or software that are central to the research but not yet described in published literature, software must be made available to editors and reviewers. We strongly encourage code deposition in a community repository (e.g. GitHub). See the Nature Portfolio guidelines for submitting code & software for further information.

Data

Policy information about availability of data

All manuscripts must include a data availability statement. This statement should provide the following information, where applicable:

- Accession codes, unique identifiers, or web links for publicly available datasets
- A description of any restrictions on data availability
- For clinical datasets or third party data, please ensure that the statement adheres to our policy

All sequencing data generated as a part of this study, support the findings of this study are openly available in GEO repository NCBI and are available with the accession number: GSE193338, GSE193333, GSE193332, and GSE193462
Figures 3, 4, 5, 6, 7 have associated raw data
There is no restriction on data availability

Human research participants

Policy information about studies involving human research participants and Sex and Gender in Research.

Reporting on sex and gender	N/A
Population characteristics	N/A
Recruitment	N/A
Ethics oversight	N/A

Note that full information on the approval of the study protocol must also be provided in the manuscript.

Field-specific reporting

Please select the one below that is the best fit for your research. If you are not sure, read the appropriate sections before making your selection.

☒ Life sciences ☐ Behavioural & social sciences ☐ Ecological, evolutionary & environmental sciences

For a reference copy of the document with all sections, see nature.com/documents/nr-reporting-summary-flat.pdf

Life sciences study design

All studies must disclose on these points even when the disclosure is negative.

Sample size	Sample sizes for ChIP-seq, RNA-seq, ATAC seq, ScRNA seq Western blot measurement were chosen empirically with following-up estimates of representation sizes, confidence level, standard deviation and experimental error with sample size re-adjustment.
Data exclusions	No data were excluded from analysis
Replication	To verify the reproducibility of experiments enough replicates were done to fit the data within the confidence range
Randomization	The samples were allocated due to the genotypes of genetically modified mice
Blinding	Blinding was non-applicable to the study since there was no need to avoid selection bias

Reporting for specific materials, systems and methods

We require information from authors about some types of materials, experimental systems and methods used in many studies. Here, indicate whether each material, system or method listed is relevant to your study. If you are not sure if a list item applies to your research, read the appropriate section before selecting a response.

Materials & experimental systems

n/a	Involved in the study
☐	☒ Antibodies
☐	☒ Eukaryotic cell lines
☒	☐ Palaeontology and archaeology
☐	☒ Animals and other organisms
☒	☐ Clinical data
☒	☐ Dual use research of concern

Methods

n/a	Involved in the study
☐	☒ ChIP-seq
☒	☐ Flow cytometry
☒	☐ MRI-based neuroimaging

Antibodies

Antibodies used

anti-UCP1 (Abcam, Cat# ab10983) (1:1000)
anti-β-actin (Santacruz, Cat# sc-47778)(1:10000)
anti-H3K27ac (Abcam, ab4729).(5 ug/ ChIP reaction)
Anti-c-Jun antibody (Abcam, ab32137) (1:1000) (5 ug/ ChIP reaction)
Anti-FRA2 antibody (Abcam, ab216838) (1:1000)
Anti-FRA-2 Antibody (Millipore) MABS1261 (1:1000)(5 ug/ ChIP reaction)
Anti-rabbit IgG, HRP-linked Antibody; Cell Signaling#7074

Validation

Several antibodies used in this study have been previously validated by the manufacturer, or in prior publications, or validated by our laboratory. anti-UCP1 (Abcam, Cat# ab10983), anti-β-actin (Santacruz, Cat# sc-47778), Anti-FRA2 antibody (ab216838) were used for western blot. anti-H3K27ac (Abcam, ab4729), Anti-c-Jun antibody (ab32137), Anti-FRA-2 Antibody MABS1261 were sued for ChIP

Eukaryotic cell lines

Policy information about cell lines and Sex and Gender in Research

Cell line source(s)

DKO mouse B6.Cg-Hmgn2tm1.1Mbus Hmgn1tm1Mbus/Mmjax (Hmgn1-/-n2-/-) MMMRC 043523-JAX

Authentication

None of these cell lines were authenticated

Mycoplasma contamination

No mycoplasma contaminations have occurred

Commonly misidentified lines
(See ICLAC register)

No commonly misidentified lines were used

Animals and other research organisms

Policy information about studies involving animals; ARRIVE guidelines recommended for reporting animal research, and Sex and Gender in Research

Laboratory animals

DKO mouse B6.Cg-Hmgn2tm1.1Mbus Hmgn1tm1Mbus/Mmjax (Hmgn1-/-n2-/-)
MMMRC
043523-JAX
C57BL/6 WT mice

Wild animals

No wild animals involved

Reporting on sex

Male mice used for most of the in vivo studies

Field-collected samples

No field collection involved

Ethics oversight

All animal maintenance and experimental procedures were performed in accordance with protocols approved by NIH Institutional Animal Care and Use Committees (IACUC)

Note that full information on the approval of the study protocol must also be provided in the manuscript.

ChIP-seq

Data deposition

☒ Confirm that both raw and final processed data have been deposited in a public database such as GEO.

☒ Confirm that you have deposited or provided access to graph files (e.g. BED files) for the called peaks.

Data access links<br>*May remain private before publication.*	https://www.ncbi.nlm.nih.gov/geo/query/acc.cgi?acc=GSE193332<br>https://www.ncbi.nlm.nih.gov/geo/query/acc.cgi?acc=GSE193333<br>https://www.ncbi.nlm.nih.gov/geo/query/acc.cgi?acc=GSE193338<br>https://www.ncbi.nlm.nih.gov/geo/query/acc.cgi?acc=GSE193462<br>https://www.ncbi.nlm.nih.gov/geo/query/acc.cgi?acc=GSE193463
Files in database submission	Each deposit has raw data and processed data
Genome browser session<br>(e.g. UCSC)	No genome browser session available

Methodology

Replicates	All RNA seq, ChIP seq, ATAC seq have three replicates. ScRNA seq has two replicates
Sequencing depth	For RNA seq libraries were paired-end sequenced (50 bp) on an Illumina HiSeq 3000.<br>The ChIP templates were sequenced at 75 bp single read length with Illumina NextSeq 500 system<br>ATAC seq libraries were paired end sequenced (50bp) on a NextSeq 500.<br>Single cell libraries were quantified, pooled, and sequenced on the NextSeq 2000 nstrument (Illumina) to a target read depth of approximately 50,000 reads per cell on average
Antibodies	anti-H3K27ac (Abcam, ab4729)
Peak calling parameters	ChIP seq peak calling: MACS2 (doi: 10.1186/gb-2008-9-9-r137) and SICER v1.1 (doi: 10.1007/978-1-4939-0512-6_5)
Data quality	CHIP seq quality control: FastQC (http://www.bioinformatics.babraham.ac.uk), PhantomPeakQualTools (doi: 10.1101/gr.136184.111), DeepTools v3.0.1 (doi:10.1093/nar/gkw257), Preseq v2.0.3 (doi: 10.1007/s40484-015-0049-7), FastqScreen v0.9.3 (doi:10.12688/f1000research.15931.2) and Kraken (doi:10.1186/gb-2014-15-3-r46) packages .
Software	RNA seq and ChIP Seq: Data were processed with CCBR Pipeliner (https://github.com/CCBR/Pipeliner)<br>RNA seq and ChIP seq: raw reads were trimmed with Cutadapt v1.18 (doi:10.14806/ej.17.1.200)<br>ChIP seq: Burrow-Wheeler Aligner BWA ver 0.7.17 (arXiv:1303.3997v2) was used to align reads to mm10 mouse genome<br>RNA seq: reads were mapped to mm10 mouse genome using STAR v2.7.0f (doi:10.1093/bioinformatics/bts635) in 2-pass mode.<br>CHIP seq quality control: FastQC (http://www.bioinformatics.babraham.ac.uk), PhantomPeakQualTools (doi: 10.1101/gr.136184.111), DeepTools v3.0.1 (doi:10.1093/nar/gkw257), Preseq v2.0.3 (doi: 10.1007/s40484-015-0049-7), FastqScreen v0.9.3 (doi:10.12688/f1000research.15931.2) and Kraken (doi:10.1186/gb-2014-15-3-r46) packages .<br>ChIP seq peak calling: MACS2 (doi: 10.1186/gb-2008-9-9-r137) and SICER v1.1 (doi: 10.1007/978-1-4939-0512-6_5)<br>ChIP seq differential binding was assessed with DiffBind (DOI: 10.18129/B9.bioc.DiffBind).<br>RNA-seq : gene-level expression was quantified using RSEM v1.3.0 (doi:10.1093/bioinformatics/bts635), with counts normalized to library size as counts-per-million.<br>RNA seq: limma-voom v3.34.5 (doi:10.1186/1471-2105-12-323) was utilized for quantile normalization and differential expression.<br>ATAC seq: Reads were mapped to the mouse genome (NCBI37/mm10) using Bowtie2<br>ATAC seq: Peak calling was performed using MACS2 using default parameters. The reads were converted to reads per thousand base pairs peak per million mapped reads (RPKM) by dividing by the total number of reads per sample.<br>ScRNA seq: Sequencing data were demultiplexed and mapped to the mouse genome (mm10: refdata-gex-mm10-2020-A) using cellranger (10X Genomics, version 6.0.0) to generate a single cell gene expression matrix.<br>ScRNA seq: filtered gene expression matrix from aggregated and read depth normalized data generated by cellranger were imported to Seurat v4 (https://satijalab.org/seurat/<br>ScRNA seq: Differential expression was calculated using every cluster against the remaining clusters using the FindAllMarkers function of the Seurat package using MAST method<br>ScRNA seq: Cell trajectory and pseudo-time analysis was performed using the Monocle R package (v2.22)
